# Pareto optimization of SPECT acquisition and reconstruction settings for ^177^Lu activity quantification

**DOI:** 10.1186/s40658-024-00667-7

**Published:** 2024-07-15

**Authors:** Johan Gustafsson, Erik Larsson, Michael Ljungberg, Katarina Sjögreen Gleisner

**Affiliations:** 1 Medical Radiation Physics, Lund, Lund University, Lund, Sweden; 2 Radiation Physics, Skåne University Hospital, Lund, Sweden

**Keywords:** Quantitative SPECT, Reconstruction, ^177^Lu, Pareto optimization

## Abstract

**Background:**

The aim was to investigate the noise and bias properties of quantitative ^177^Lu-SPECT with respect to the number of projection angles, and the number of subsets and iterations in the OS-EM reconstruction, for different total acquisition times.

**Methods:**

Experimental SPECT acquisition of six spheres in a NEMA body phantom filled with ^177^Lu was performed, using medium-energy collimators and 120 projections with 180 s per projection. Bootstrapping was applied to generate data sets representing acquisitions with 20 to 120 projections for 10 min, 20 min, and 40 min, with 32 noise realizations per setting. Monte Carlo simulations were performed of ^177^Lu-DOTA-TATE in an anthropomorphic computer phantom with three tumours (2.8 mL to 40.0 mL). Projections representing 24 h and 168 h post administration were simulated, each with 32 noise realizations. Images were reconstructed using OS-EM with compensation for attenuation, scatter, and distance-dependent resolution. The number of subsets and iterations were varied within a constrained range of the product number of iterations $\times$ number of projections $\le 2400$. Volumes-of-interest were defined following the physical size of the spheres and tumours, the mean activity-concentrations estimated, and the absolute mean relative error and coefficient of variation (CV) over noise realizations calculated. Pareto fronts were established by analysis of CV versus mean relative error.

**Results:**

Points at the Pareto fronts with low CV and high mean error resulted from using a low number of subsets, whilst points at the Pareto fronts associated with high CV but low mean error resulted from reconstructions with a high number of subsets. The number of projection angles had limited impact.

**Conclusions:**

For accurate estimation of the ^177^Lu activity-concentration from SPECT images, the number of projection angles has limited importance, whilst the total acquisition time and the number of subsets and iterations are parameters of importance.

## Background

Quantitative Single Photon Emission Computed Tomography (SPECT) forms the basis for patient-specific dosimetry in molecular radiotherapy (MRT), and as new therapeutic radiopharmaceuticals are introduced into clinical practice, the need for accurate *in vivo *activity estimation is increasing. The radionuclide ^177^Lu is used for treatments of neuroendocrine tumours (^177^Lu-DOTA-TATE) [1] and prostate cancer (^177^Lu-PSMA) [2], and has favourable imaging properties [3]. Dosimetry in MRT will allow for better understanding of the therapeutic effect and, in the long term, possibly optimization and individualization of treatments [4]. For this potential to be realized, the use of accurate dosimetry, and thus accurate quantitative SPECT, is a prerequisite.

Inaccuracy in SPECT-based activity-concentration estimation results from both systematic errors and random errors. A limitation of SPECT is its poor spatial resolution, leading to partial-volume effects (PVEs) and related systematic underestimation of the mean activity concentration in small regions with uptake above background. In principle, such systematic errors can be corrected for post reconstruction, most commonly by the application of recovery coefficients. However, such a correction will always be associated with an uncertainty that propagates to the final result. The management of PVEs is closely connected to the strategy for image segmentation, which together are often considered as the dominant sources of uncertainty for MRT absorbed-dose estimates [5–7]. Hence, it is desirable to keep systematic errors low. Random errors will result from the non-perfect repeatability of nuclear-medicine imaging, where the stochastic nature of the emission and detection of photons from radioactive decay results in noise in the acquired projections, which propagates to the reconstructed image.

There is a well-known conflict between good spatial resolution, i.e. low systematic errors, and good noise properties, i.e. low random errors, for modern iterative SPECT reconstruction methods, e.g. the ML-EM and OS-EM algorithms [8–10]. A high number of image updates favours good resolution but tends to deteriorate the signal-to-noise ratio (SNR). However, the exact object-formation and noise properties of the reconstruction algorithms are complex with dependence both on the object itself, its environment, and the model of the projection-formation process used in the reconstruction [11–13]. Likewise, the rate of convergence of region-based activity estimates also depends on properties related to the activity-distribution itself, in combination with the properties of the reconstruction algorithm and the projection-formation model. A key concept in this context is that SPECT for activity estimation is a fundamentally different task than SPECT for patient diagnosis [14]. Hence, experiences from diagnostic imaging may not be directly transferable to MRT dosimetry. The optimization of quantitative SPECT for MRT dosimetry is thus a non-straightforward multi-objective problem where the two opposing requirements of low systematic errors and low random errors have to be compromised.

Since low bias (absence of systematic errors) and good precision (low random errors) are opposing objectives for SPECT-based activity-concentration estimation, a single optimal setting for acquisition and reconstruction can in general not be found. Instead, a range of settings can be considered optimal, in the sense that no other choice of parameters is able to achieve a lower bias without deteriorating precision or vice versa. These points are referred to as the Pareto front. Pareto analysis is an established technique for representation of problems with opposing objectives and has been found useful in a range of applications, e.g., engineering and treatment planning in external-beam radiotherapy [15, 16], and similar ideas have previously been applied for nuclear medicine imaging in, for example, the comparison of reconstruction methods for diagnostic imaging [17].

Even if noise is typically not a major source of uncertainty for MRT dosimetry, its effect will become substantial for imaging at very low SNRs [18, 19]. There is currently a strive to reduce imaging times for clinical dosimetry [20, 21], resulting in increased patient comfort and decreased risk of movement artefacts. The need for shorter acquisition protocols has become particularly pressing for SPECT of ^177^Lu-PSMA, where it is necessary to acquire multiple bed positions to obtain adequate axial coverage, resulting in extensive acquisition times unless the time per bed-position is reduced.

Shorter acquisition protocols can be achieved by changing either the number of projections or the time per projection. A reduced time per projection will deteriorate the SNR in the projections, in turn propagating to a poorer SNR in the reconstructed image. On the other hand, a reduced number of projections may lead to angular under-sampling. This may both affect how noise propagates to the reconstructed image, and expand the null-space of the reconstruction problem, meaning that the number of source distributions that are compatible with measured projections increases and may then also increase systematic errors. These considerations also tie back to the relatively complex noise properties of iterative SPECT reconstruction, and the propagation of errors from the acquisition in projection space will be different depending on the reconstruction settings and projection-formation model used [11, 12, 22].

Current guidelines for SPECT-based MRT dosimetry provide advice on how the number of projections should be selected. For example, according to MIRD 23 [23] angular under-sampling is minimized by setting the number of projections at least equal to the matrix size (e.g., 128 projections for a $128\times 128$ matrix). Guidelines focusing on dosimetry for ^177^Lu-labelled radiopharmaceuticals describe the usual range of number of projections for ^177^Lu SPECT as 60–120 [24], or recommend between 60 and 120 projections but also state that fewer projections might yield satisfactory results [3]. However, little empirical justification is given for these recommendations. Theoretical principles for angular sampling for tomography in general have been presented [25, 26], but these are not necessarily relevant for the specific task of quantitative SPECT. Likewise, the problem of how to distribute a total acquisition time over different projections has been extensively studied for small-animal imaging by Li and Meng [27], but the acquisition geometry in that study, involving pinhole collimation, differs substantially from a clinical scenario.

All things considered, there is a need for improved understanding of the relationship between the number of projections, time per projection and resulting errors in quantitative SPECT, in particular in view of the clinical need to reduce examination times. Hence, the aim of this study is to study noise and bias properties of SPECT-based ^177^Lu activity-concentration estimation, using Pareto optimization, with respect to the number of projections, time per projection, and number of updates and subsets in the iterative tomographic reconstruction.

## Methods

### Physical-phantom measurements

Two phantoms filled with ^177^Lu were prepared. Phantom A was a NEMA IEC Body PET phantom with 6 spheres (in order of ascending volume: 0.52 mL, 1.15 mL, 2.72 mL, 5.61 mL, 11.7 mL, and 26.9 mL), henceforth referred to as spheres 1 to 6, in a non-radioactive background. A stock solution with ^177^Lu was prepared to fill the spheres, adding EDTA to prevent sticking to the walls. An activity meter and balance with traceability to primary standard were used for preparation, and decay correction was performed using a half-life of 6.647 d [28].The sphere activity-concentration was ${5.80}\,\rm{MBq}\,\rm{mL}^{-1}$ at the start of acquisition. Phantom B was a cylindrical Jaszczak phantom filled with a uniform activity concentration of ${22.8}\,\rm{kBq}\,\rm{mL}^{-1}$.

One hundred twenty projections were acquired over ${360}^{\circ }$ using a GE Discovery 670 SPECT/CT system (GE Healthcare, Haifa, Israel) with 5/8” crystals equipped with medium-energy collimators. The acquisition for phantom A used 180 s per projection and the acquisition for phantom B used 90 s per projection. List-mode data were saved for further off-line processing.

#### Bootstrapping

Data for phantom A were retrieved from the list file as floating-point matrices (non-disclosure agreement with GE Healthcare), and binned into 1 s time bins in $128\times 128$ matrices with pixel size $4.42\times 4.42\,\mathrm {mm^{2}}$ for a 15 % energy window centred at 208 keV. Data sets consisting of 20, 24, 30, 40, 60, and 120 projections, distributed over $360^\circ$ with constant angular sampling, were created. For each set, projections corresponding to a total acquisition time of 40 min, 20 min, and 10 min, were generated using bootstrapping with 32 realisations per setting by pixel-wise drawing of random time-bins with replacement. Note that acquisition times refer to virtual time, i.e., the time required for a single detector to acquire the corresponding projection data. For a dual-headed camera, the physical time is half the virtual time excluding time for detector movement.

#### SPECT reconstruction

Tomographic images were reconstructed using OS-EM with compensation for attenuation, scatter using the ESSE method, and distance-dependent geometric spatial resolution [29]. For a given number of projections, all subset settings supported by the reconstruction program were explored. A prerequisite was that the number of subsets was an even integer divisor of the number of projections (e.g., 2, 4, 6, 10, 12, 20, and 30 for an acquisition with 60 projections). The maximum number of iterations was set such that the product between number of projections and number of iterations did not exceed 2400. For example, an acquisition with 60 projections allowed for a maximum of 40 iterations, resulting in the maximum number of updates ranging from 80 to 1200, corresponding to 2 to 30 subsets, respectively. The upper limit on the product of number of projections and number of iterations was considered a substitute for the total reconstruction time, i.e., that a fixed maximum total computational time was available for reconstruction and that the time per iteration was roughly proportional to the size of the system matrix. Reconstructed images were saved after each iteration. The reconstruction schemes used for the different number of projections are summarized in Table 1. Note that the presented values are maximum number of iterations and updates and that images for all number of iterations up to those were included.

**Table 1:** Reconstruction schemes employed for different number of projections with respect to maximum number of iterations, number of subsets, and resulting maximum number of updates

Num. proj.	Max. num. iter.												
120	20	Num. subs.	2	4	6	8	10	12	20	24	30	40	60
		Max. upd.	40	80	120	160	200	240	400	480	600	800	1200
60	40	Num. subs.	2	4	6	10	12	20	30				
		Max. upd.	80	160	240	400	480	800	1200				
40	60	Num. subs.	2	4	8	10	20						
		Max. upd.	120	240	480	600	1200						
30	80	Num. subs.	2	6	10								
		Max. upd.	160	480	800								
24	100	Num. subs.	2	4	6	8	12						
		Max. upd.	200	400	600	800	1200						
20	120	Num. subs.	2	4	10								
		Max. upd.	240	480	1200								

#### Calibration

The list-mode data for phantom B were used to generate projection sets with the same range of number of projections as for phantom A, but using the full 90 s acquisition time without any bootstrapping. Tomographic images were reconstructed using the same range of settings as for phantom A.

For determination of the calibration factor, a large cylindrical volume-of-interest (VOI) of 375 mL was defined in the centre of the phantom in the SPECT image. The mean signal-concentration in the VOI was divided by the activity-concentration determined from phantom preparation. Since the reconstruction program included normalization of the voxel values to total acquisition time, the result was a calibration factor such that when applied to an image yielded voxel values in terms of activity. This procedure was undertaken for each projection set and reconstruction setting, to obtain the calibration factor for the respective reconstruction of the physical-phantom measurement.

### Monte Carlo simulations

An XCAT phantom [30] coupled to a pharmacokinetic model of ^177^Lu-DOTA-TATE [31] was used as basis. Three tumours were included with volumes of 2.8 mL, 8.9 mL, and 40.0 mL, for tumour 1, tumour 2, and tumour 3, respectively. The SIMIND Monte Carlo program [32] was used to simulate SPECT projections at 24 h and 168 h post injection with the same parameters as for the bootstrapped physical-phantom measurements (phantom A). The tumour activity-concentrations, derived from the model, were ${1.9}\,\rm{MBq}\,\rm{mL}^{-1}$ and ${0.75}\,\rm{MBq}\,\rm{mL}^{-1}$ for images corresponding to 24 h and 168 h post injection, respectively. The full source was defined by the pharmacokinetic model as specified in Brolin et al. [31]. For liver, spleen, left kidney, and right kidney, the activity concentrations were ${0.14}\,\rm{MBq}\,\rm{mL}^{-1}$, ${0.51}\,\rm{MBq}\,\rm{mL}^{-1}$, ${0.34}\,\rm{MBq}\,\rm{mL}^{-1}$, and ${0.45}\,\rm{MBq}\,\rm{mL}^{-1}$ at 24 h post injection and ${0.040}\,\rm{MBq}\,\rm{mL}^{-1}$, ${0.12}\,\rm{MBq}\,\rm{mL}^{-1}$, ${0.051}\,\rm{MBq}\,\rm{mL}^{-1}$, and ${0.068}\,\rm{MBq}\,\rm{mL}^{-1}$ at 168 h post injection. Concentrations for kidneys refer to the average over cortex, medulla, and pelvis. One hundred twenty projections were simulated for a nominal time of 1 s per projection and datasets for 20, 24, 30, 40, 60, and 120 projections were created by removing intermediate projections. A large number of histories were run to create essentially noise-free projections (i.e., residual Monte Carlo noise negligible compared with the Poisson noise of a real measurement). The projections were scaled such that they corresponded to an injected activity of 7400 MBq, accounting for excretion via the pharmacokinetic model and physical decay of ^177^Lu from the time of injection to the respective imaging time-point, and total acquisition times of 40 min, 20 min, and 10 min. Thirty-two noise realisations per acquisition setting were generated by addition of Poisson-distributed noise. Tomographic images were reconstructed as for the physical-phantom measurements.

#### Calibration

Calibration was performed similarly as for the physical phantom measurements. Projections were simulated for a cylinder (radius 10.8 cm, length 18.6 cm) filled with ^177^Lu. Reconstruction-setting specific calibration factors were determined by defining a large cylindrical VOI (1953 mL) in the centre of the phantom and dividing the image signal concentration with the activity concentration defined by simulation input. Since the aim of these calibration simulations was not to study effects of noise on calibration factors, Poisson noise was not added to projections before reconstruction, i.e., mimicking a calibration measurement with long acquisition time. These calibration factors were applied to simulated data, for the respective reconstruction scenario.

### Evaluation

#### Physical-phantom measurements

Spherical VOI masks with the same volumes as the NEMA phantom spheres (within the volume of one voxel) were defined. The activity concentration estimated from images was compared with the corresponding concentration estimated from phantom preparation. The absolute value of the mean relative error was calculated as $\left| {\bar{C}}_{\rm{est}}/C_{\rm{ref}}-1\right| ,$ ${\bar{C}}_{\rm{est}}$ denoting the mean estimated activity concentration over bootstrap realizations and $C_{\rm{ref}}$ the reference activity concentration. The coefficient of variation (CV) over bootstrap realizations was calculated as $\rm{CV}=s/{\bar{C}}_{\rm{est}}$ with *s* denoting the standard deviation in estimated activity concentration. CV was calculated for each combination of acquisition and reconstruction settings.

Scatter plots of CV and the absolute mean relative error were studied for the different sphere sizes and total acquisition times. The Pareto fronts, i.e., the set of points where the absolute mean relative error could not be reduced without increasing the CV or vice versa, were identified. Occasionally, due to instability in the reconstruction at high noise levels, the estimated mean activity concentration became zero, making the CV undefined [33]. These data points were excluded from further analysis.

#### Monte Carlo simulations

Volumes of interest for the tumours were defined based on the digital phantom masks, which were geometrically transformed to the SPECT-image coordinate system. The mask transformation and binarization was made so as to preserve the mask volume as closely as possible (within the volume of a single voxel). This was achieved by considering the analytical coordinates of the voxels represented by a mask and the SPECT image, respectively, and including voxels for which the spatial overlap was the highest, until the original volume was reached. The mean relative error and the CV over the noise realizations were studied for each combination of acquisition and reconstruction settings.

Scatter plots of CV and the absolute mean relative error were studied for the three tumours at 24 h and 168 h p.i., respectively, and different total acquisition times. Pareto fronts were constructed similarly as for the physical-phantom measurement.

## Results

### Phantom measurements

**Fig. 1:**
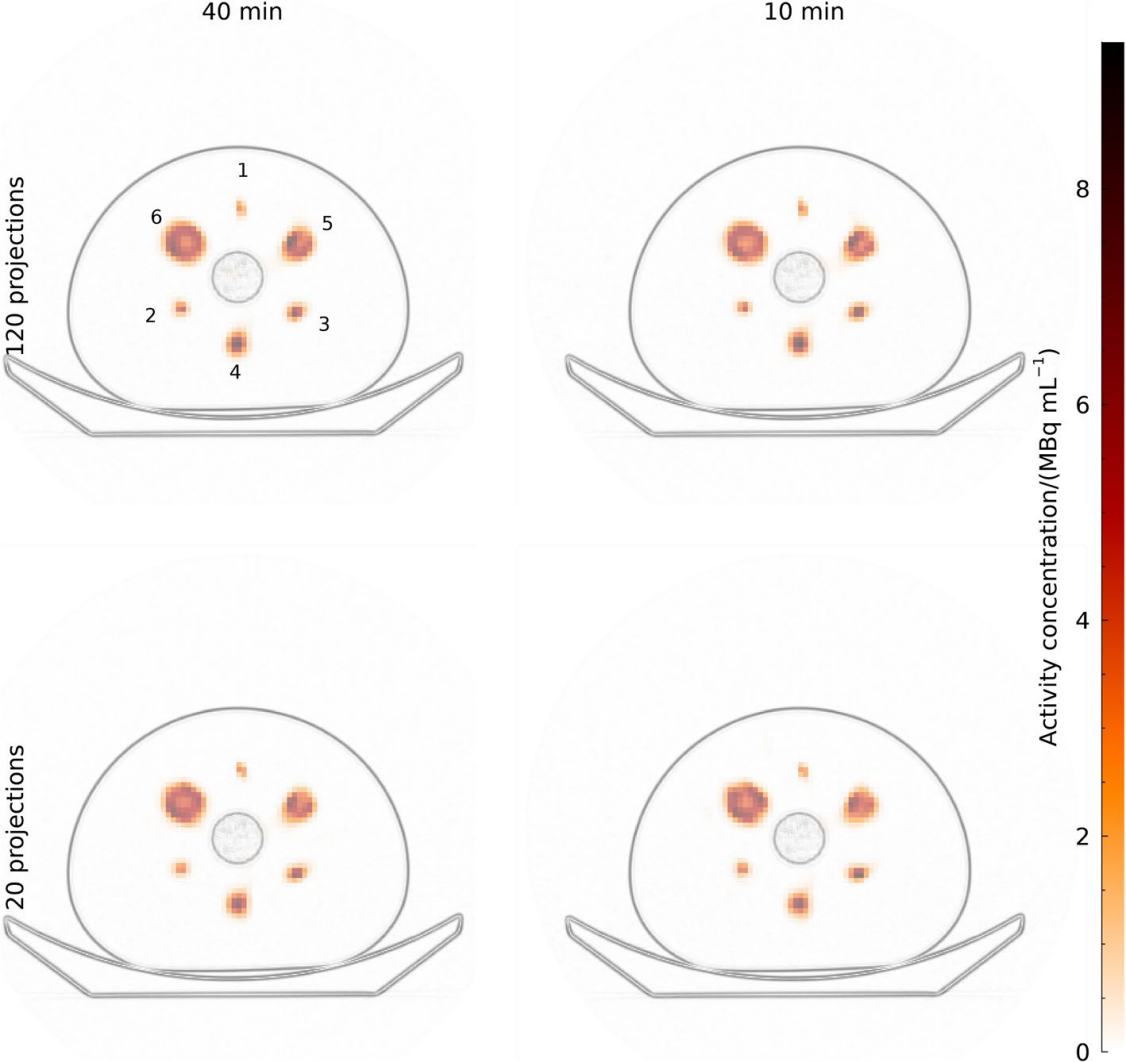
Examples of reconstructed SPECT images of the NEMA phantom. The SPECT images are shown overlaid on a high-pass filtered CT images. All images are for 96 updates with 5 angles per subset. Sphere numbers are indicated in the image for acquired with 120 projections for 40 min

Examples of reconstructed images are shown in Fig. 1 for the extreme cases of 120 projections and 20 projections with total acquisition times of 40 min and 10 min. There is a tendency for spheres to be resented as slightly non-spherical shapes for the examples with 20 projections, but generally the differences between images are subtle. A plot of calibration factors for different reconstruction settings is presented in the supplement (Fig. S1).

**Fig. 2:**
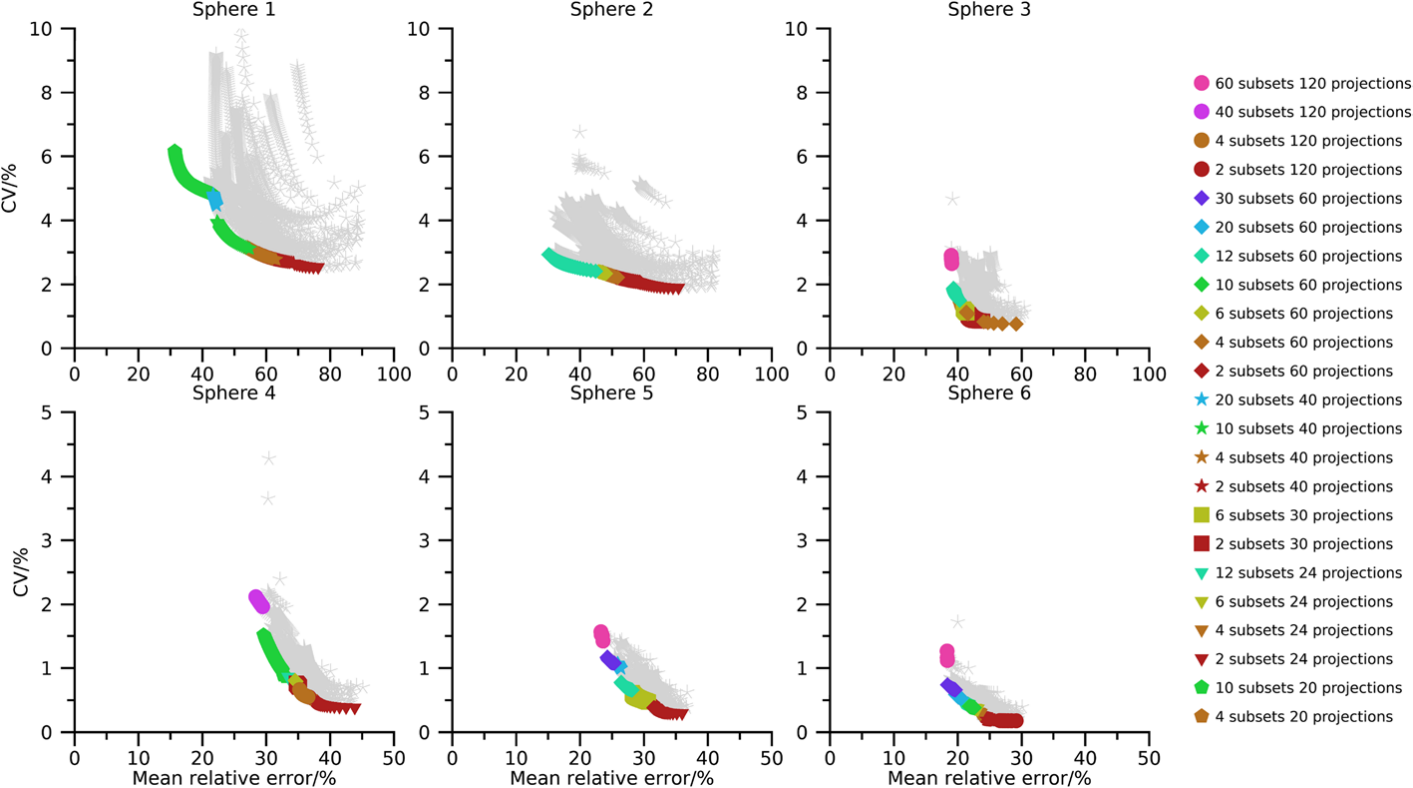
Absolute relative error and CV for NEMA spheres with total acquisition time 40 min. Coloured points indicate Pareto optimal data points while grey asterisks indicate non-Pareto optimal data points. Ordinates have been capped at 10 % for spheres 1 to 3 and 5 % for spheres 4 to 6

**Fig. 3:**
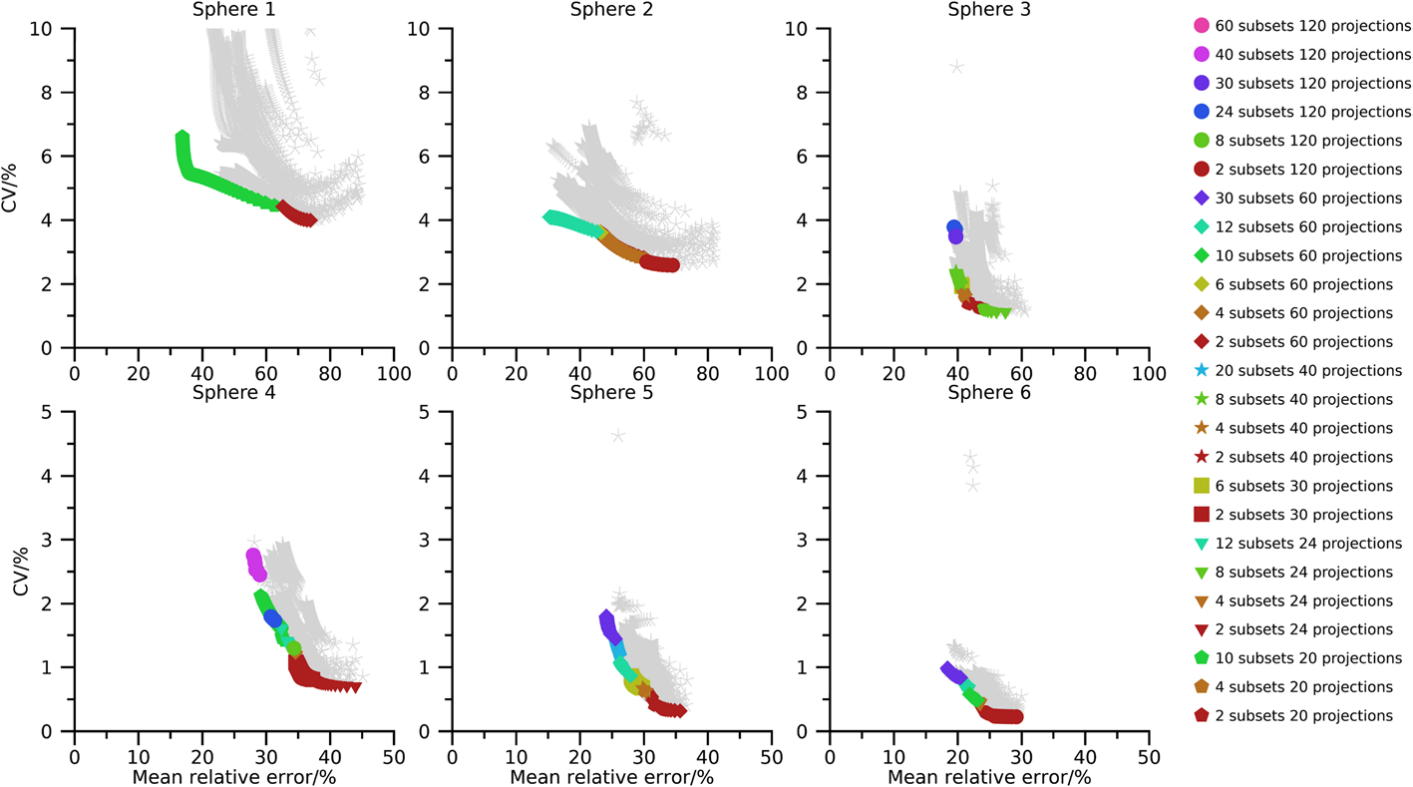
Absolute relative error and CV for NEMA spheres with total acquisition time 20 min. Coloured points indicate Pareto optimal data points while grey asterisks indicate non-Pareto optimal data points. Ordinates have been capped at 10 % for spheres 1 to 3 and 5 % for spheres 4 to 6

**Fig. 4:**
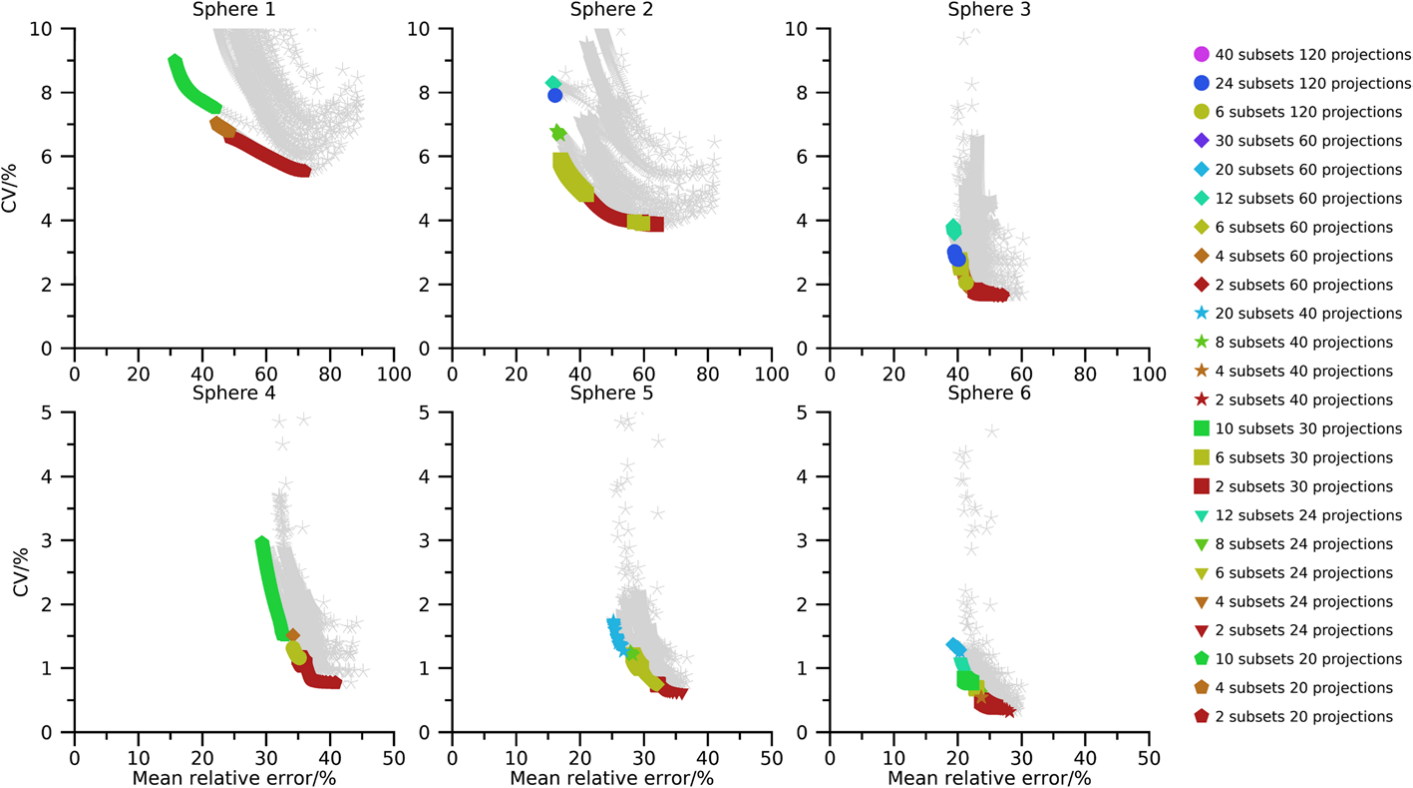
Absolute relative error and CV for NEMA spheres with a total acquisition time of 10 min. Coloured points indicate Pareto optimal data points while grey asterisks indicate non-Pareto optimal data points. Ordinates have been capped at 10 % for spheres 1 to 3 and 5 % for spheres 4 to 6

Pareto fronts of activity-concentration estimates are shown in Figs. 2, 3, and 4 for 40 min, 20 min, and 10 min total (virtual) acquisition time, respectively. At least two iterations and 16 updates are required for an activity-estimate to be included in the plots. By comparison of Figs.2, 3, and 4 it is seen that as the total acquisition time decreases, the CV tends to increase while the mean error remains more stable, introducing a vertical shift of the Pareto fronts as a function of total acquisition time. The CV also tends to decrease as the sphere volume increases, and the three largest spheres are typically associated with CVs below 5 %, even when the total acquisition time is as short as 10 min. A wide range of number of updates are present in the Pareto fronts with the minimum and maximum being 16 and $1\,200$. Points associated with low CVs but comparably high mean errors mainly result from OS-EM reconstructions with only two subsets (dark red colour in Figs. 2, 3, and 4), corresponding to a long acquisition time per subset. Note that for a fixed total acquisition time and a fixed number of subsets, the time per subset is constant even if the number of projections changes. It is noteworthy in this context that these low-noise high-bias points do not show a clear pattern with respect to total number of projections. For example, both 24 projections (triangles) and 120 projections (circles) are represented in this part of the Pareto front. Plots of the absolute mean relative error and CV for a fixed number of updates as function of number of projections and time per subset are presented in the supplemental material (Figs. S3–S20). These plots reveal the consistent trend that an increased time per subset is associated with improved noise properties (i.e., lower CVs), while the number of projections has less systematic effects on bias and precision. The most consistent trend is that a large number of projections, combined with a low number of updates, increase the CV. Correspondingly, settings associated with low bias but comparably high CV are the opposite. These Pareto-optimal points are associated with a large number of subsets relative to the total number of projections for the SPECT acquisition, e.g., 60 subsets (cerise in Fig. 2, 3, 4) for 120 projections and 12 subsets (turquoise) for 24 projections. Noteworthy, a wide range of total number of projections are represented, with both 20 projections (pentagons) and 120 projections (circles) being represented.

### Monte Carlo simulations

**Fig. 5:**
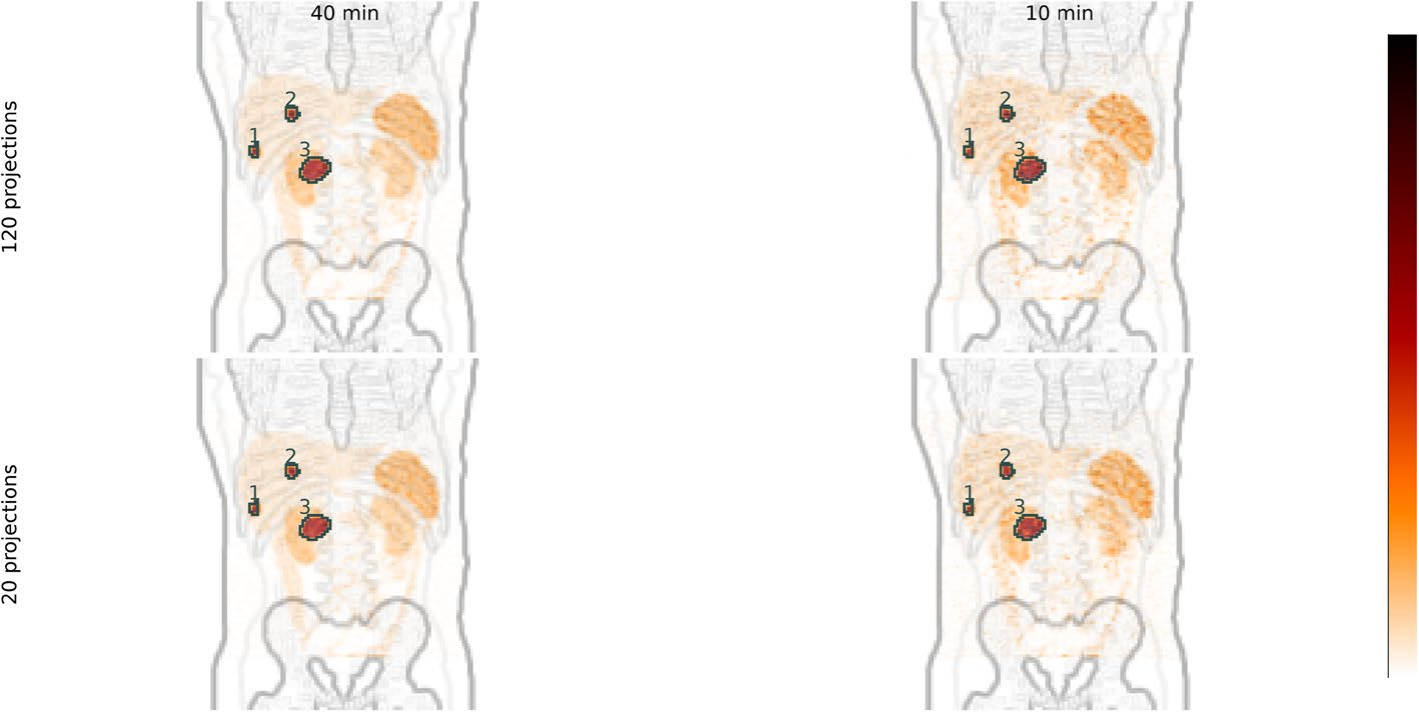
Examples of reconstructed images of the simulated XCAT phantom for different total acquisition times, distributed between different number of projections. All images are for 96 updates with 5 angles per subset

Examples of maximum-intensity projections of reconstructed SPECT images for the extreme cases of images are shown in Fig. 5. As noted, there is little visual difference between examples acquired with 120 projections and 20 projections. A plot of calibration factors for different reconstruction settings is presented in the supplement (Fig. S2).

**Fig. 6:**
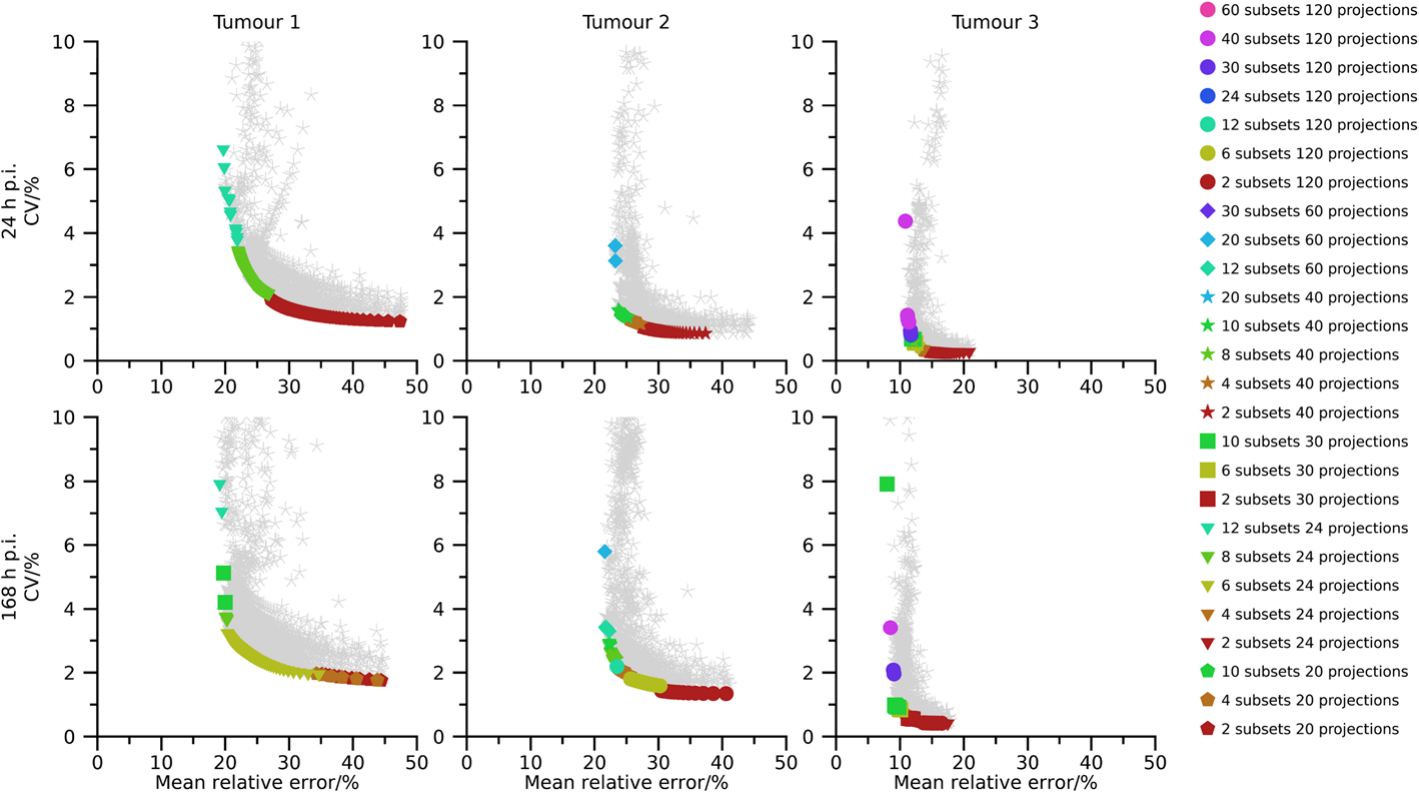
Absolute relative error and CV for simulated tumours with a total acquisition time of 40 min. Coloured points indicate Pareto optimal data points while grey asterisks indicate non-Pareto optimal data points. Ordinates have been capped at 10 %

**Fig. 7:**
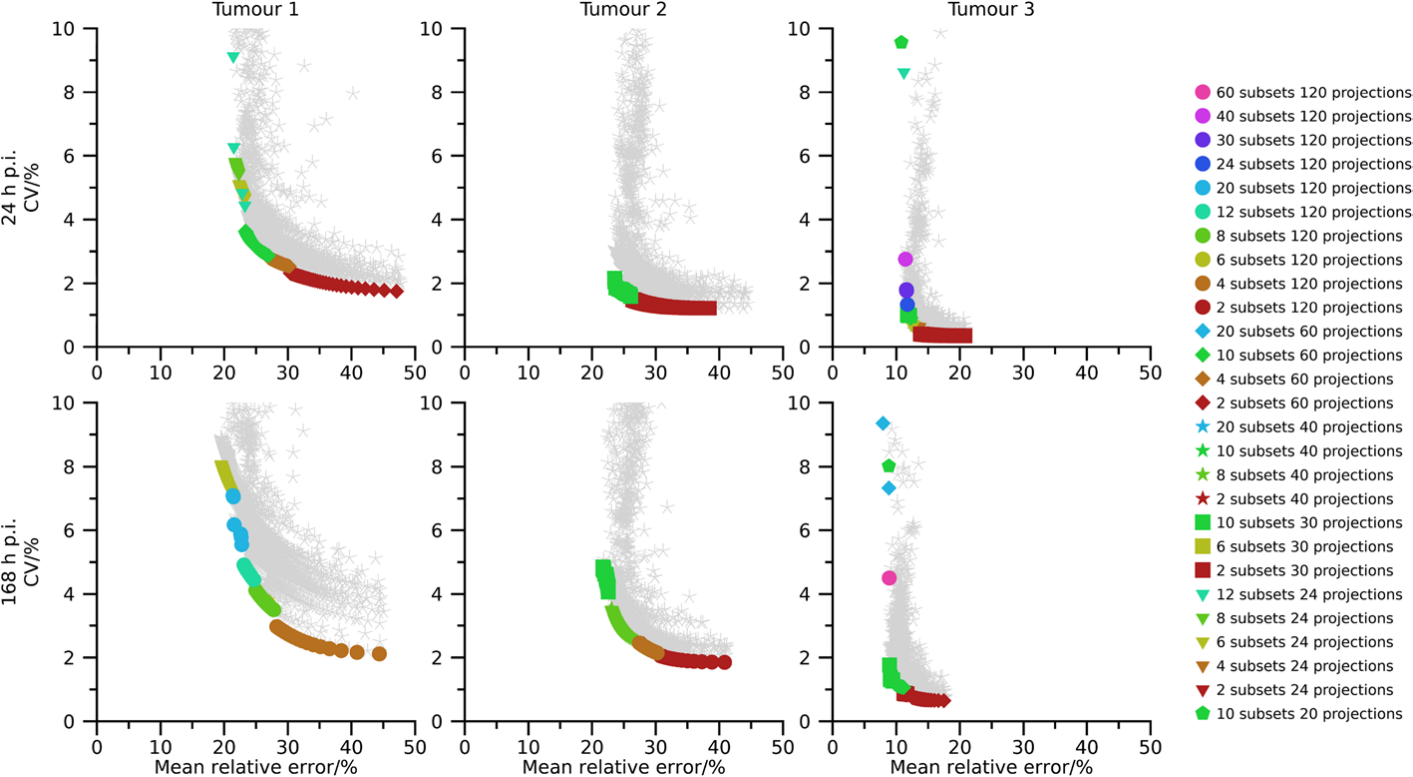
Absolute relative error and CV for simulated tumours with a total acquisition time of 20 min. Coloured points indicate Pareto optimal data points while grey asterisks indicate non-Pareto optimal data points. Ordinates have been capped at 10 %

**Fig. 8:**
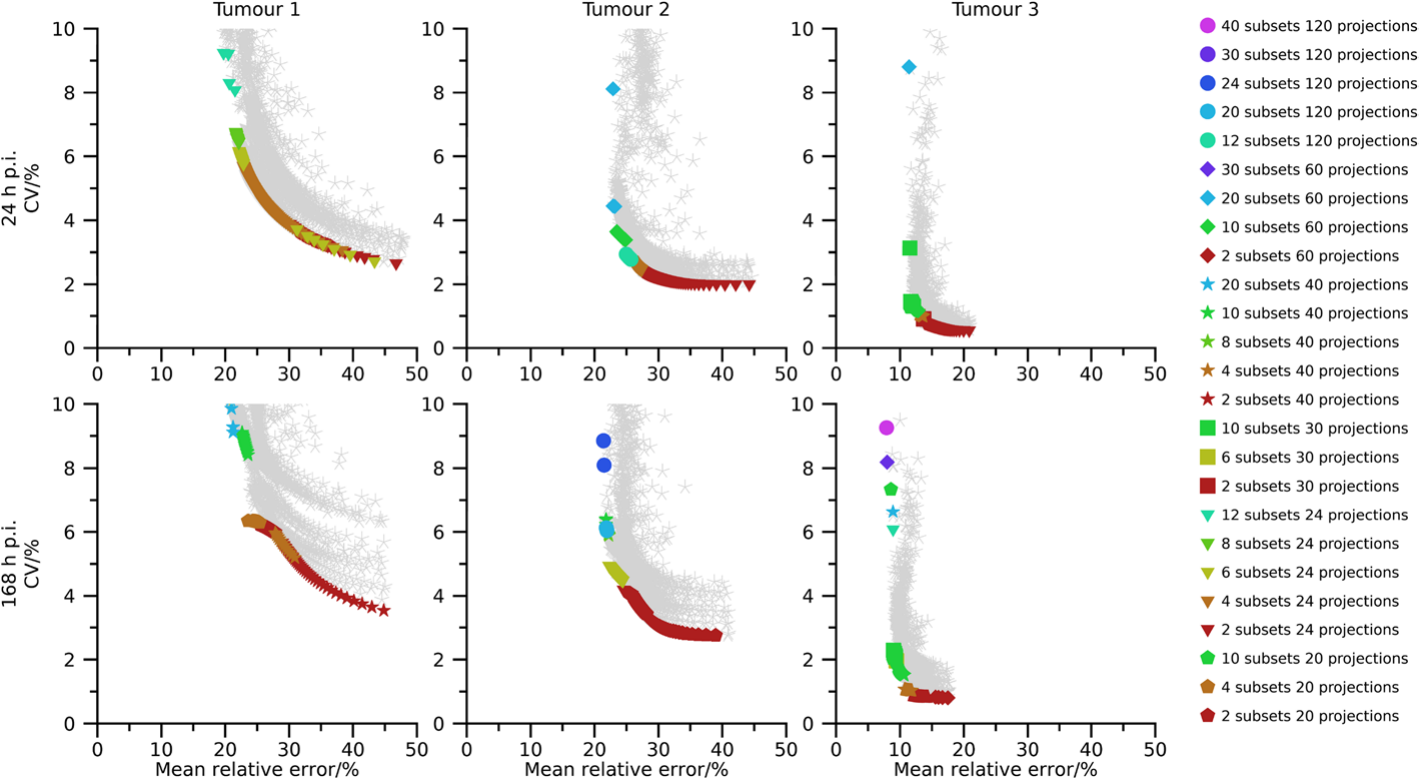
Absolute relative error and CV for simulated tumours with a total acquisition time of 10 min. Coloured points indicate Pareto optimal data points while grey asterisks indicate non-Pareto optimal data points. Ordinates have been capped at 10 %

Pareto fronts for activity-concentration estimates for tumour 1 to tumour 3 are shown in Figs. 6, 7, and 8 for total acquisition times of 40 min, 20 min, and 10 min, respectively. At least two iterations and 16 updates are required for an activity-estimate to be included in the plots. Compared with the physical-phantom measurements there is a wider range of CVs present in data, but otherwise results correspond well. A wide range of number of updates were present in the Pareto fronts, with the minimum and maximum being 16 and $1\,200$, a shorter acquisition time results in an upward shift in the Pareto-fronts, Pareto-optimal points with low CV but high bias are associated with a long time per subset, and Pareto-optimal points associated with a low bias but high CV are associated with a large number of subsets. Again, a wide range of total number of projections are present in the Pareto fronts and this parameter had little systematic impact when studying the bias and dispersion separately (supplemental material).

## Discussion

We have systematically studied the noise and bias properties for SPECT-based activity-concentration estimation of ^177^Lu, of importance for dosimetry in MRT. A general trend in results is that the most important parameters are associated with the reconstruction (number of iterations and subsets) and the total acquisition time, whilst the number of projections have a less systematic and often small effect on bias and precision. In other words, the total acquisition time can be distributed over few or many projection angles, with essentially the same resulting activity-concentration estimation properties. This information is useful as it removes one parameter in the optimization of SPECT-based activity-concentration estimation, and appropriate attention can be given to those that have a higher impact. The underlying philosophy behind the evaluation is that the quality of an image should be judged from its ability to perform the task for which it was acquired [14]. That is, for quantitative SPECT, the primary measure of image quality is the capability of the image for accurate activity-concentration estimation.

Bootstrapping has previously been demonstrated a powerful tool for studying the noise-properties of tomographic imaging [34–37], but has not been extensively applied to the problem of quantitative SPECT in MRT. The advantage of this technique is that it allows to use experimental data for studying the precision of derived activity concentrations without having to rely on a projection model, as opposed to the Monte Carlo simulated images. The physical phantom measurement and Monte Carlo simulations are complementary in this sense. The physical measurements did not rely on the extensive model assumptions associated with a simulation approach. On the other hand, the physical measurements were based on a stylized phantom, whilst the simulated images considered a more relevant geometry that also included a complex background. As opposed to the papers by Buvat and Riddell [34] and Buvat [35] we considered all pixels in the projections as statistically independent. This was motivated by the low count rate of the phantom study (approximately ${700}\,\rm{s}^{-1}$ in the main window), making non-linear effects, such as dead-time, negligible. However, the bootstrapping did not assume the number of counts in a pixel to be Poisson distributed. Indeed, the projection time-bins used in the bootstrapping had been corrected for linearity and uniformity and hence did not consist of integer values, which, by definition, caused deviation from a Poisson distribution.

Another reason for the use of both measured and simulated geometries was to cover a wide range of activity concentrations. The activity concentration of the NEMA phantom was in the higher end of what would be expected clinically [38]. The main reason for this high concentration was the need to have a sufficient total activity in the phantom, combined with a limited total active volume. To some extent, this problem was addressed by investigating a range of acquisition times, but direct translation of results to the clinic is not necessarily straight-forward. The simulated anthropomorphic computer phantom, based on a pharmacokinetic model, is more representative of patients both in terms of tumour activity concentrations and total activity.

The use of Pareto optimization is a well-known tool for optimization problems with opposing objectives. This perspective is useful for SPECT since there is a well-known conflict between bias and noise for tomographic techniques. In the context of MRT dosimetry, the effect of noise has been less studied than bias, and has often been argued to be negligible compared with the uncertainties associated with bias correction [5–7]. Although this is true for many cases, there are situations where the effect of noise increases in importance, e.g., for image-based estimation of low activities [19], or imaging with short acquisition times [18]. Currently, there is a clinical interest to reduce image acquisition times in ^177^Lu-based MRT, but at some point this endeavour will be hindered by image noise, in particular for small objects such as tumours. Our data show that large regions are relatively insensitive to noise with respect to activity-concentration estimation (Figs. 2, 3, 4, 6, 7 and 8), with a low-to-moderate CV also for the shortest acquisition time of 10 min. For smaller objects, such as the smallest sphere (0.5 mL) and the smallest tumour (2.8 mL), the CV is substantially higher. However, in practice these estimates are associated with considerable bias that limits the capability of quantification [33]. The limiting factor is not so much related to the compensation of partial-volume effects as such, as indicated by the mean relative errors in Figs. 2, 3, 4 and 6, 7, and 8, but the difficulties involved in VOI definition for small regions [33, 39].

The trend that Pareto-optimal points with low CV but high bias tend to result from reconstructions having a long time per subset can be intuitively understood from the perspective that longer time per subset means that more data are included in every update of the source-estimate, which in turn should lead to a better noise behaviour. On the contrary, Pareto-optimal points with lower bias but higher CV are associated with a large number of subsets in relation to the number of acquired projections. This difference can be understood from the well-known property of OS-EM reconstruction, that spatial resolution, and in turn bias of activity-concentration estimates, improves with an increased number of updates, whilst noise, associated with CV in the current context, increases.

There are a number of potential problems associated with substantially reducing the number of projections in tomographic imaging, which is illustrated by the small, but potentially distracting, deviations from spherical shapes in Fig. 1. There is substantial literature available on how a limited number of projections affects the solvability of the reconstruction problem [25, 26]. A reduced number of projections will inevitably increase the null-space of the projector, which in turn may increase bias. However, the problem has been less studied in the context of SPECT-based activity-concentration estimation, in which the primary aim is to estimate the average in one or several regions rather than giving a good representation of the whole object. A major limitation of clinical SPECT imaging is its poor spatial resolution, which in itself causes source distributions to be located in the projector null-space. Hence, the theoretical question at hand is which factor is the primary source of information loss in the projection operation, and there is reason to believe that the main limitation is the spatial resolution of the camera rather than the angular sampling of the tomographic acquisition. We believe that this is the main reason for the lack of systematics in how the number of projections affects the noise-versus-bias trade-off, i.e., angular sampling is simply not the limiting factor with respect to the deterministic properties of the reconstructed image, at least not for the range of projections investigated in this study. At some point, the angular sampling will become a relevant factor that, but this does not seem to be the case even for as few as 20 projections which is considerably less than typically used for clinical applications, at least for the kind of geometries covered within this study.

One major limitation of the current study is the limited number of noise realizations (32) for each acquisition setting, both for the physical and digital phantom, leading to an uncertainty in the mean relative error and the CV. This is a minor limitation for the mean error, since the standard deviation is substantially lower than the mean, and thus the standard-error of the mean substantially lower than the mean. In contrast, under the assumption of normality, the relative standard-error of the standard deviation for a sample size of 32 is more than 10 % [40], and there is thus non-negligible room for data points to move vertically in the Pareto plots. The main challenge in using a larger sample size is the increased computational burden and the storage of reconstructed images for further analysis, for which reasons a larger sample size was not deemed feasible.

An issue related to the limited sample size is the problem of outliers and the stability of SPECT reconstructions at different settings. For the physical-phantom measurement, a total of 6 out of 182, 400 SPECT images (0.003 %) where lost during processing (for unknown reasons), and out of the $1\,900$ generated settings 115 (6 %) caused a median activity concentration of zero over all noise realizations or at least one outlier activity concentration, defined as deviating more than 50 % from the median, for at least one sphere over all noise realization. These outliers occurred mainly for few angles per subset (i.e., a large number of subsets relative to the number of projections) and the majority was for the shortest total acquisition time (10 min). Corresponding results for simulated data were 261 and 311 for activity distributions at 24 h p.i. and 168 h, p.i., respectively, again mainly for images reconstructed with few angles per subset. Outlier data-points were considered part of the distribution of activity-concentration estimates, and were thus included in the analysis. An exception was made for cases where all noise realizations resulted in an estimate of zero, in which case the CV is undefined.

Aiming for low CV and low bias in quantitative SPECT is intuitively reasonable. In practical MRT dosimetry the bias is usually compensated for using explicit partial-volume correction, and further analysis would include the bias and precision after such correction. However, such analyses would be tightly linked to the particular methods used for image segmentation and partial-volume correction. Likewise, the relevant quantity for MRT dosimetry is the time-integrated activity concentration rather than the activity concentration at a single time-point. The integration over time tends to act averaging with respect to random errors and will partly mitigate random errors associated with single time points. Still, understanding the properties of the basic input (the activity concentration) to this curve-fitting and integration is important in its own right, not the least for understanding of the propagation of errors through the calculation and for a better understanding of the whole procedure.

One particularity of the SPECT reconstruction used in this study is the use of model-based scatter compensation with the ESSE method [29]. The availability of model-based scatter-compensation methods has increased during recent years by the adoption of Monte Carlo based reconstruction [20, 41–43], but still scatter is often estimated using window based techniques [44, 45]. Since window-based scatter compensation methods are themselves affected by noise, the accuracy of such methods should be affected by the balance between the number of projections and the time per projection. The use of window-based methods may thus lead to a larger effect of the time per projection on bias and precision of activity-concentration estimates. A reasonable hypothesis would be that a longer time per projection, associated with fewer projections for a fixed total acquisition time, should lead to a more accurate scatter compensation and thus more accurate activity-concentration estimates. However, this would also be affected by a number of other parameters, e.g., energy window-widths and the use of filtering of scatter estimates, and would warrant further investigation. Furthermore, the number of updates studied is larger than typically used for non-regularized SPECT reconstruction. However, we find that a large number of updates is necessary in order to achieve stable activity-concentration estimates [46] This is, in turn, caused by the non-linear nature of OS-EM reconstruction and the slow convergence of reconstructions including resolution compensation, with convergence rate being dependent on the source-geometry studied [47]. Of note, this is based on the premise that the aim is to estimate mean activity-concentrations in a region, for which intra-regional non-uniformities induced by, e.g., noise or Gibbs artefacts, are less relevant.

In addition to the well known phenomenon that bias is improved whilst precision is worsened by an increased number of updates, our results demonstrate that: *a*) A shorter total acquisition time deteriorates precision, but has a small effect on bias. *b*) Precision is favoured by a long acquisition time per subset, and *c*) For a fixed acquisition time, a wide range of number of projections can result in Pareto optimal activity-concentration estimates. That is, it is of minor importance if the total acquisition time is distributed over few or many projections, whilst the bias and precision of activity-concentration estimates are strongly dependent on the settings of the OS-EM reconstruction. Furthermore, results suggest that acquisition times can be shortened without major effects on bias and that the increased imprecision can partly be mitigated by decreasing the number of subsets while keeping the number of updates constant.

## Conclusions

The bias and precision of quantitative ^177^Lu SPECT primarily depend on the total acquisition time in combination with the number of updates and subsets in the OS-EM tomographic reconstruction. The distribution of the total acquisition time over few or many projections has little impact on bias and precision. Good precision is favoured by a long time per subset, which has the potential to compensate for the reduced number of detected counts when decreasing total acquisition time.

### Supplementary Information

Supplementary Material 1.

## Data Availability

The datasets generated or analysed during the current study are not publicly available due the associated large storage requirements but are available from the corresponding author on reasonable request.

## Abbreviations

CV: Coefficient of variation
MRT: Molecular radiotherapy
PVE: Partial-volume effect
SNR: Signal-to-noise ratio
SPECT: Single photon emission computed tomography
VOI: Volume of interest
