# Supplementary material for: Pareto optimization of SPECT acquisition and reconstruction settings for 177Lu activity quantification
Source: EJNMMI Phys. 2024 Jul 15;11:62. doi: 10.1186/s40658-024-00667-7 (PMC11247071; doi:10.1186/s40658-024-00667-7)
Supplement: Supplementary file 1 — Supplementary Material 1. [file 40658_2024_667_MOESM1_ESM.pdf]

# Supplement to Pareto optimization of SPECT acquisition and reconstruction settings for $^{177}$Lu activity quantification

Johan Gustafsson[1,*], Erik Larsson[2], Michael Ljungberg[1], and Katarina Sjögreen Gleisner[1]

1. Medical Radiation Physics, Lund, Lund University, Lund, Sweden

2. Radiation Physics, Skåne University Hospital, Lund, Sweden

## 1 Calibration

Measured SPECT calibration factor as function of number of updates is shown in figure S1. The calibration factors stabilize after the first few updates and demonstrate a modest dispersion across number of projections and subsets. When imposing the restriction of a minimum of two iterations and 16 updates, the calibration factors range from $8.14\,\mathrm{s}^{-1}\,\mathrm{MBq}^{-1}$ to $8.48\,\mathrm{s}^{-1}\,\mathrm{MBq}^{-1}$.

Simulated SPECT calibration factors as function of number of updates are shown in figure S2. As for the physical phantom measurements, calibration factors stabilize after the first few updates. When imposing the restriction of a minimum of two iterations and 16 updates, the calibration factors range from $8.00\,\mathrm{s}^{-1}\,\mathrm{MBq}^{-1}$ to $8.17\,\mathrm{s}^{-1}\,\mathrm{MBq}^{-1}$.

## 2 Mean relative error and CV as function of number of projections

Plots of the mean relative errors for the NEMA measurements as function of number of projections are shown in figure S3 to figure S5 for total acquisition times of 40 min, 20 min, and 10 min. Plots are shown for 200 updates, 600 updates and 800 updates. Corresponding results for CV are shown in figure S6 to figure S8. The monotonicity of the mean error and CV as function of number of iterations was characterized by Spearman's correlation coefficient. A coefficient for which $p < 0.05$ was considered significant. For the mean error, the correlation was significantly negative for seven and significantly positive for three out of 18 cases (spheres and total acquisition times) when employing 200 updates, four and three out of 18 when employing 400 updates, and two and two out of 18 when employing 800 updates. Significant positive correlations were mainly demonstrated for the small spheres while negative correlations were mainly for the larger spheres. For negative correlations, the effect size was typically a few percentage points. For CV, there was a significant positive correlation for 13 out of 18 cases, eight out of 18 cases, and seven out of 18 cases for 200, 400, and 800 updates, respectively.

Plots of the mean relative error for the Monte Carlo simulated images are shown in figure S9 to figure S11 for total acquisition times of 40 min, 20 min, and 10 min. Plots are shown for 200 updates, 600 updates and 800 updates. Corresponding results for CV are shown in figure S12 to figure S14. For the mean errors, correlations were significantly negative for seven out of nine cases, seven out of nine cases , and one out of nine cases for 200, 400, and 800 updates, respectively, at 24 h p.i. Corresponding results for 168 h p.i. were five out of nine cases, four out of nine cases, and zero out of nine cases. With respect to CV, correlations were significantly positive for six out of nine cases, one out of nine cases, and zero out of nine cases at 24 h p.i., and three out of nine cases, one out of nine cases, and zero out of nine cases at 168 h p.i.

## 3 Coefficient of variation as function of time per subset

Plots of the CV for the NEMA measurements as function time per subset are shown in figure S15 to figure S17 for total acquisition times of 40 min, 20 min, and 10 min. Plots are shown for 200 updates, 600 updates and 800 updates. Spearman's correlation coefficients are indicated in the plots. Seventeen out of 18 cases had a significant negative correlation when employing 200 updates, twelve out of 18 cases for 400 updates, and five out of 18 cases for 800 updates. Note that the ranges of time per subset are larger for lower number of updates.

Plots of the CV for the Monte Carlo simulated images are shown in figure S18 to figure S20 for total acquisition times of 40 min, 20 min, and 10 min. Plots are shown for 200 updates, 600 updates and 800 updates. At 24 h p.i., nine out of nine cases had a significant negative correlation when employing 200 updates, eight out of nine cases for 400 updates, and four out of nine cases for 800 updates. Corresponding results for 168 h p.i. were nine out of nine, seven out of nine, and one out of nine. Note that the ranges of time per subset are larger for lower number of updates.

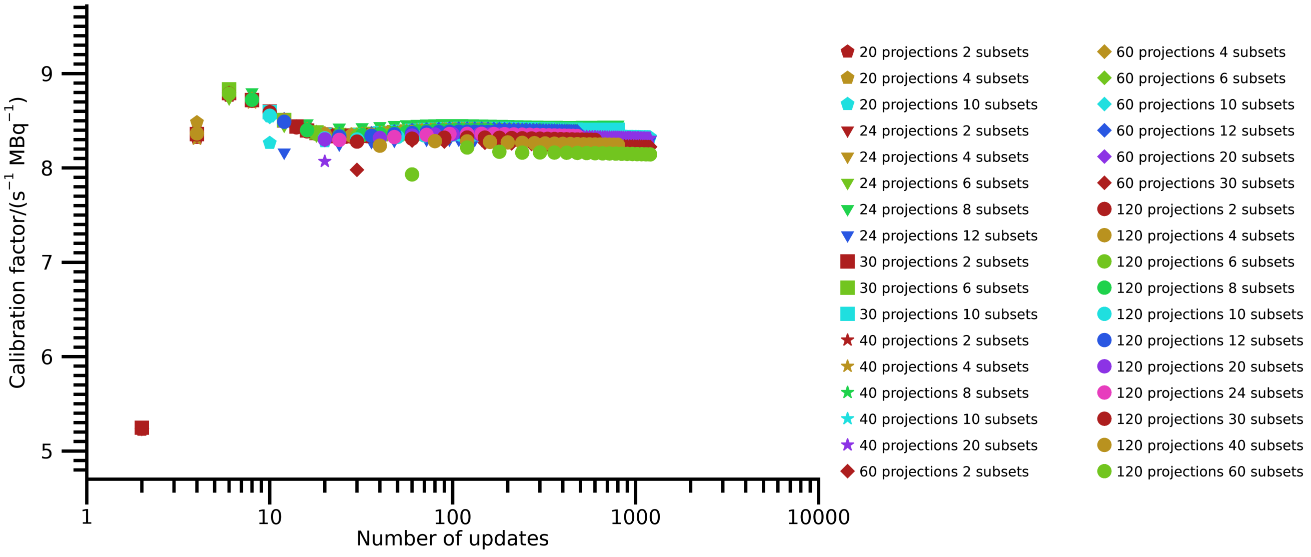


Figure S1: Measured SPECT calibration factors for different acquisition and reconstruction settings as function of number of image updates.

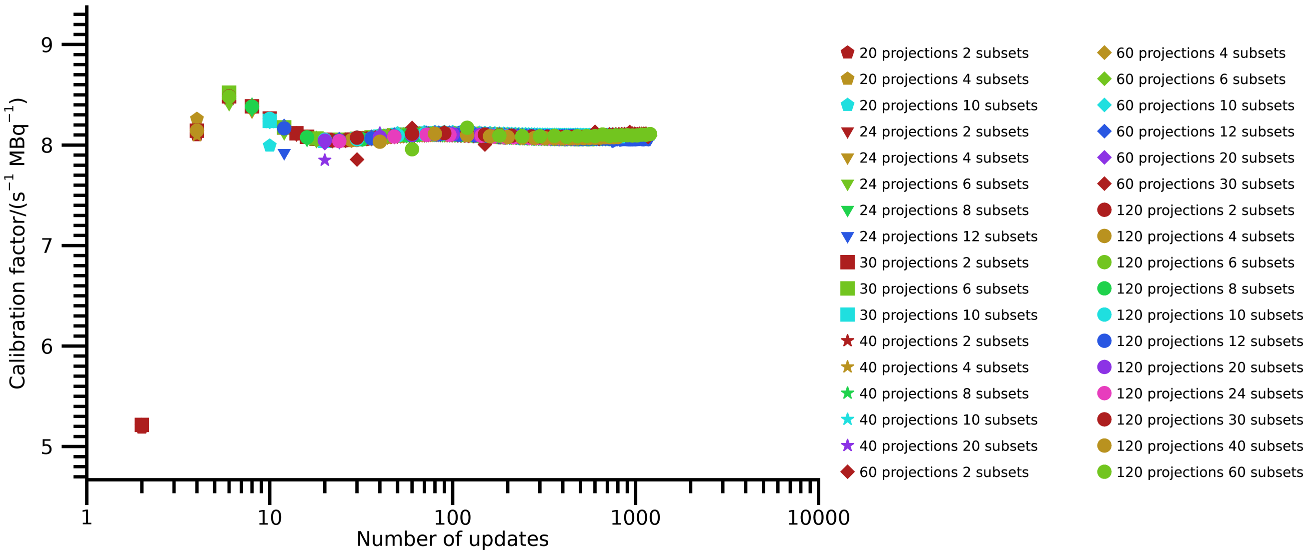


Figure S2: Simulated SPECT calibration factors for different acquisition and reconstruction settings as function of number of image updates.

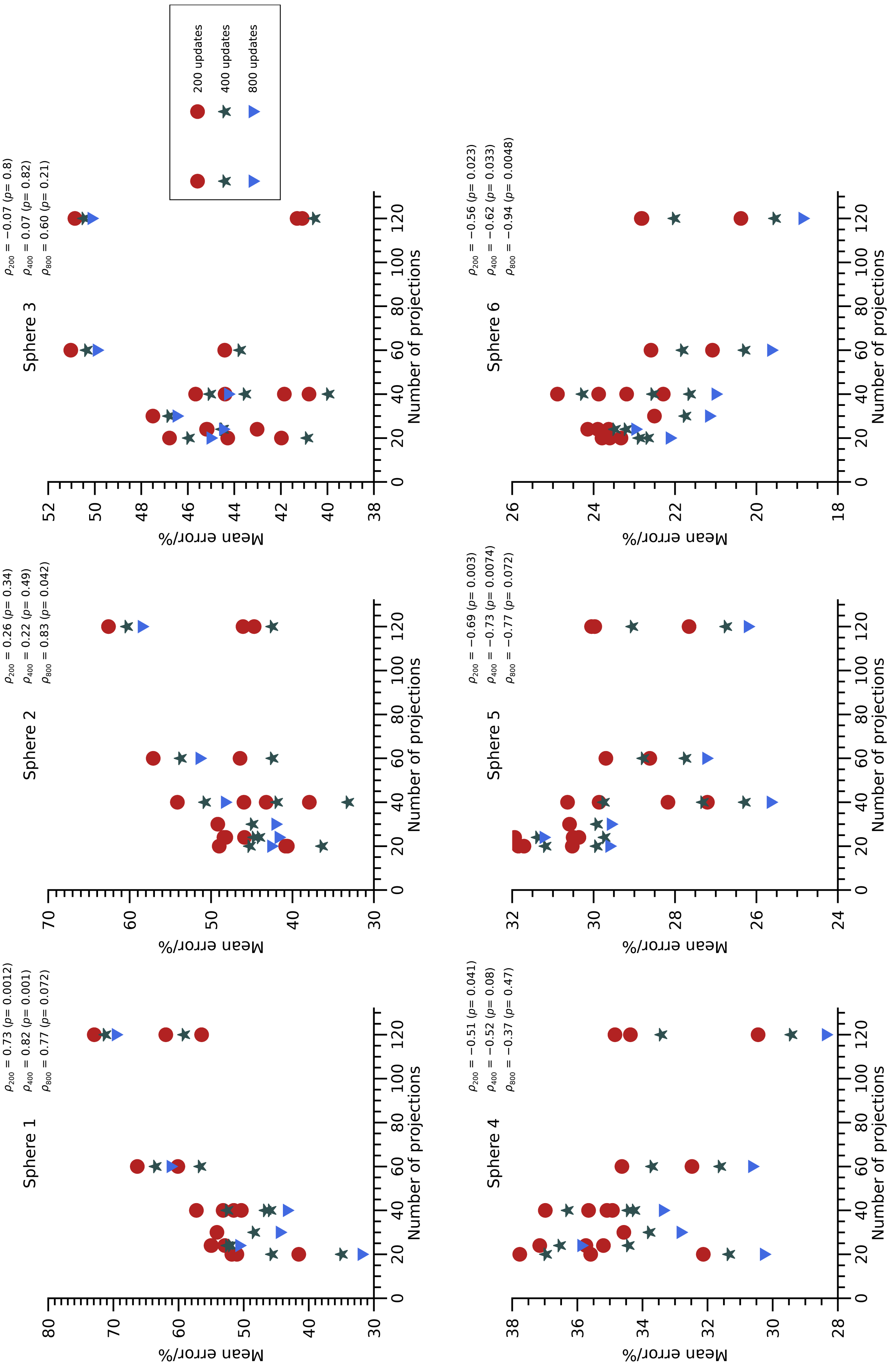


Figure S3: Absolute mean relative error as function of number of projections of the NEMA phantom measurements with a total acquisition time of 40 min. Results are shown for 200, 400, and 800 updates. Note that the ordinate range is individual for each plot.

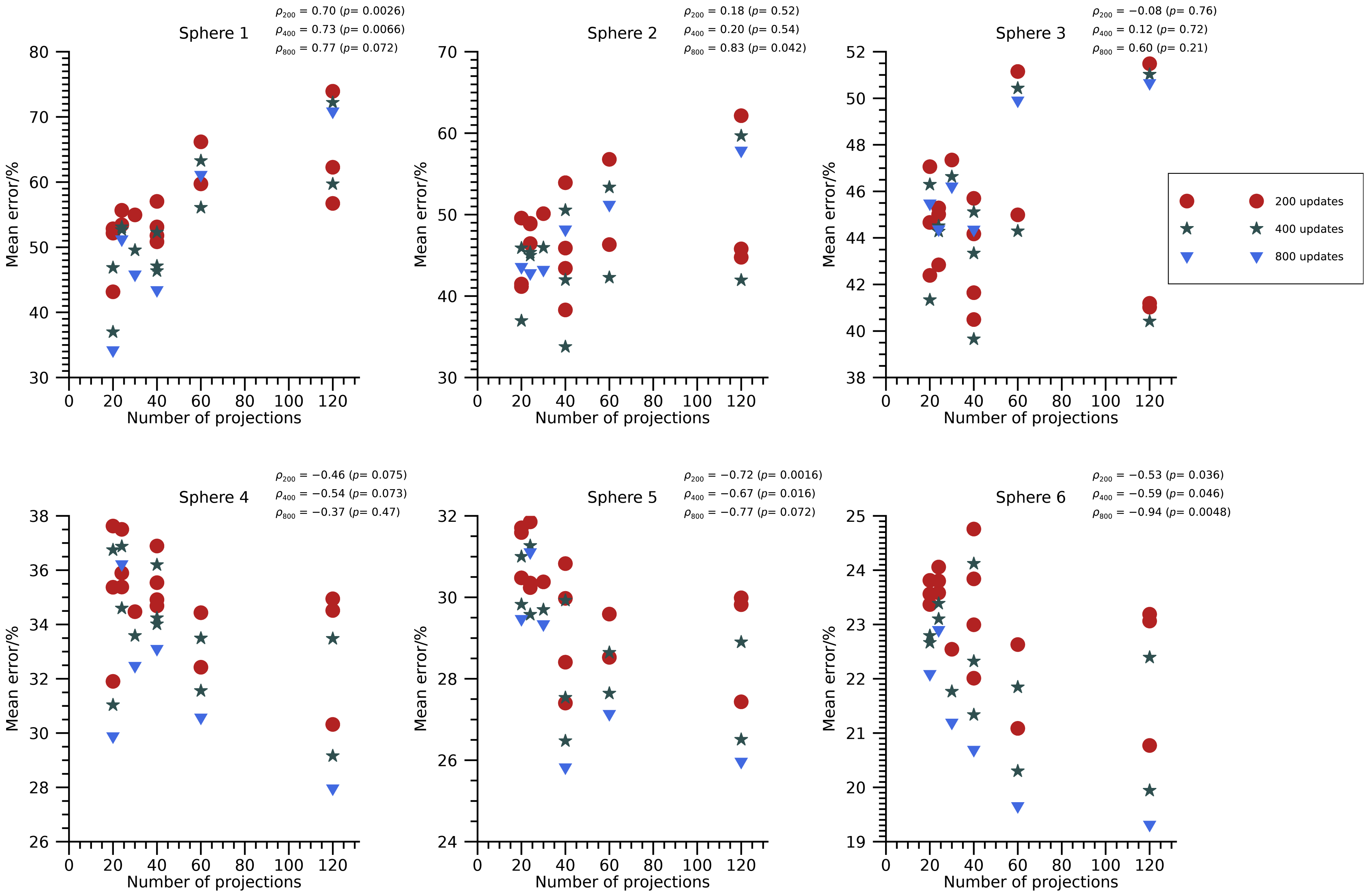


Figure S4: Absolute mean relative error as function of number of projections of the NEMA phantom measurements with a total acquisition time of 20 min. Results are shown for 200, 400, and 800 updates. Note that the ordinate range is individual for each plot.

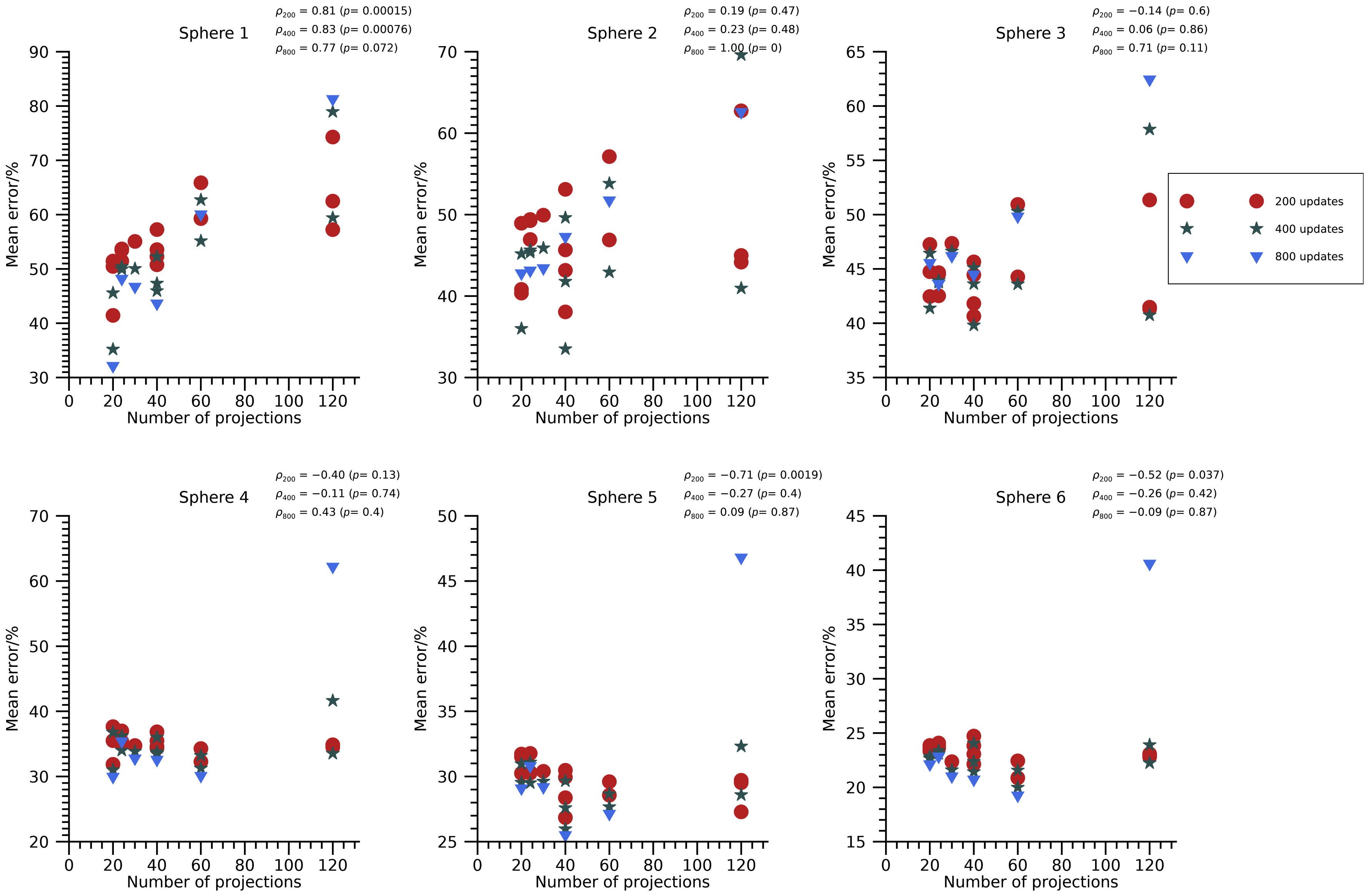


Figure S5: Absolute mean relative error as function of number of projections of the NEMA phantom measurements with a total acquisition time of 10 min. Results are shown for 200, 400, and 800 updates. Note that the ordinate range is individual for each plot.

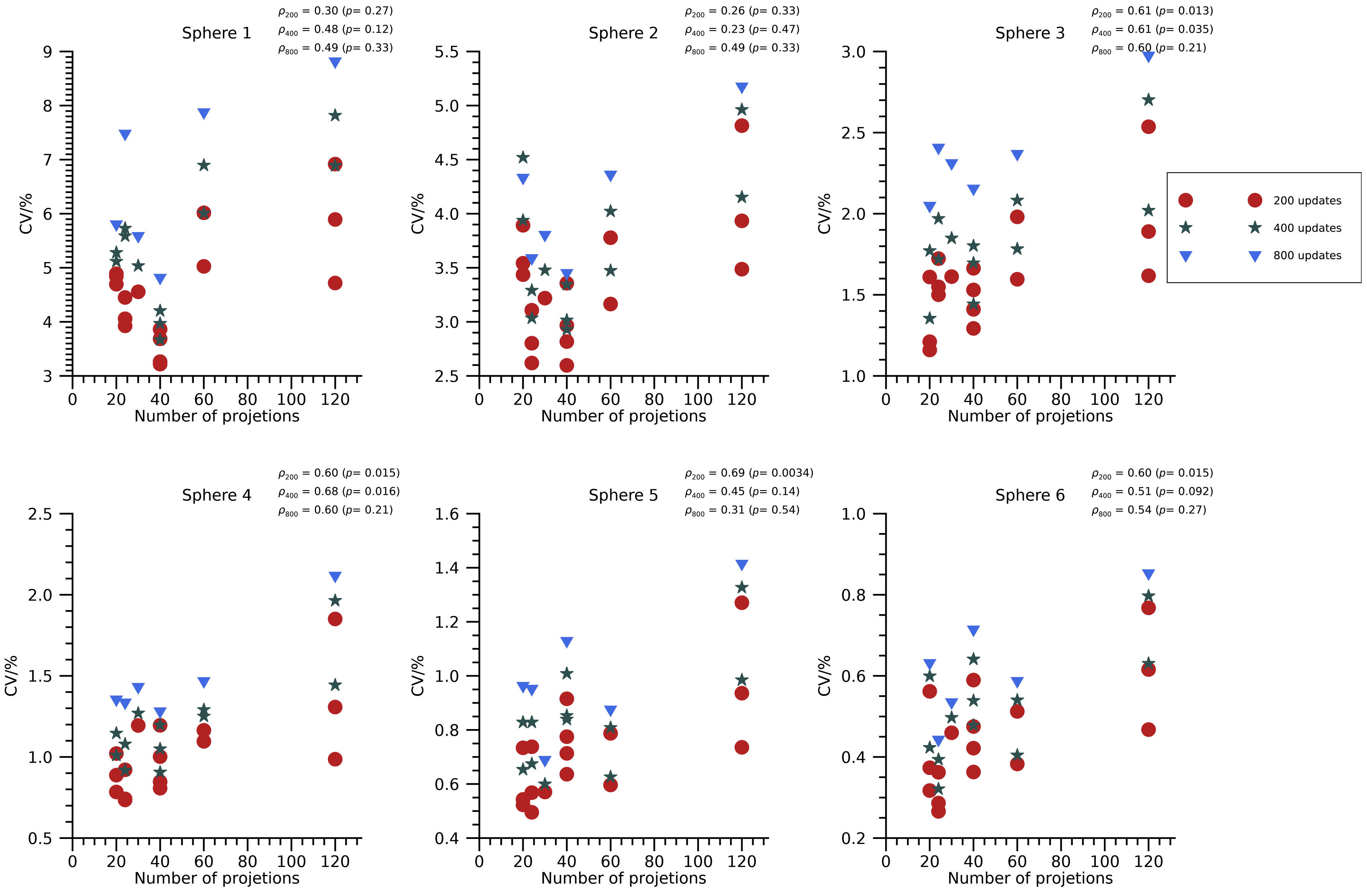


Figure S6: Coefficient of variation error as function of number of projections of the NEMA phantom measurements with a total acquisition time of 40 min. Results are shown for 200, 400, and 800 updates. Note that the ordinate range is individual for each plot, but, if necessary, has been capped at 20 %.

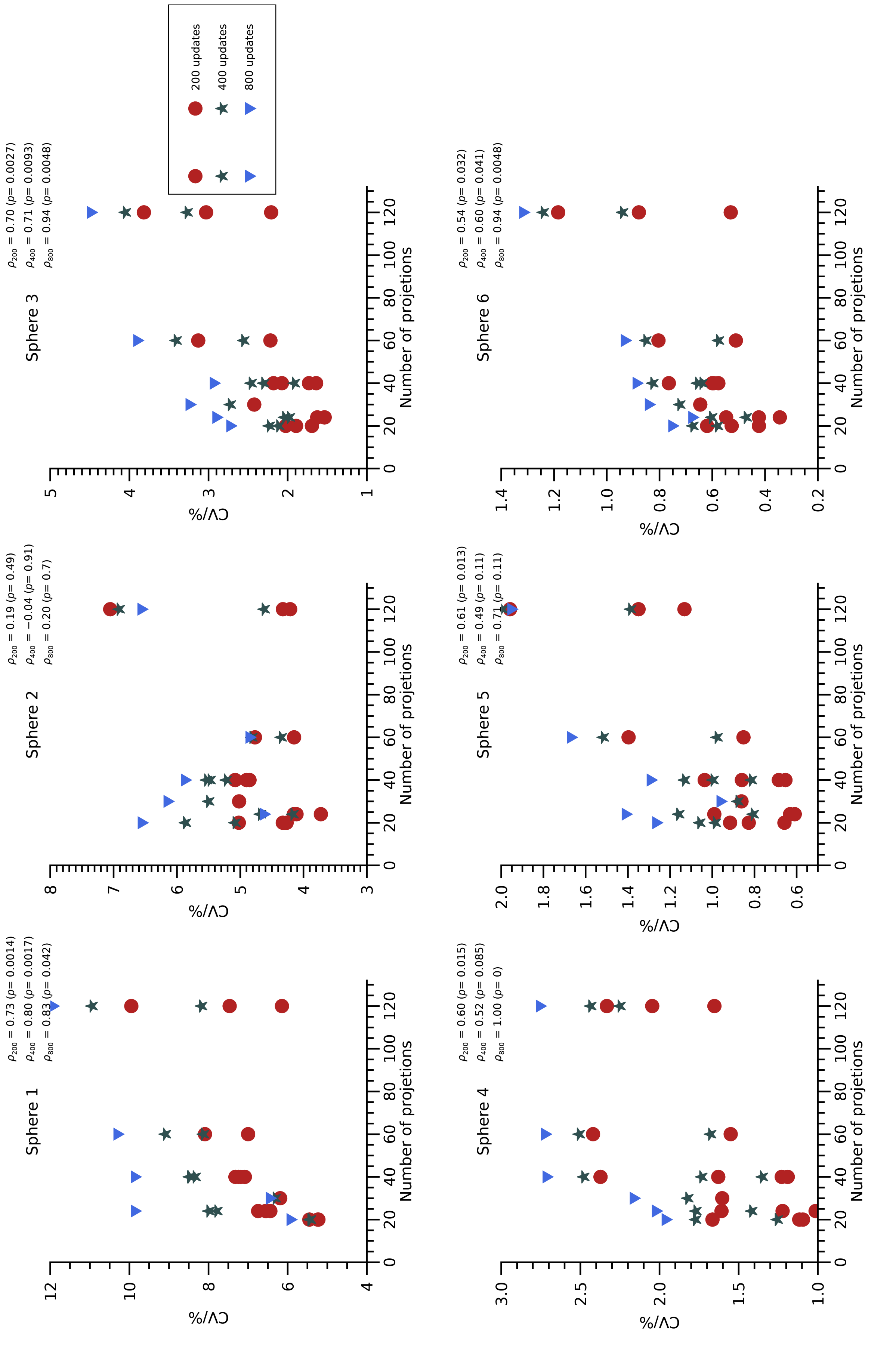


Figure S7: Coefficient of variation error as function of number of projections of the NEMA phantom measurements with a total acquisition time of 20 min. Results are shown for 200, 400, and 800 updates. Note that the ordinate range is individual for each plot, but, if necessary, has been capped at 20 %.

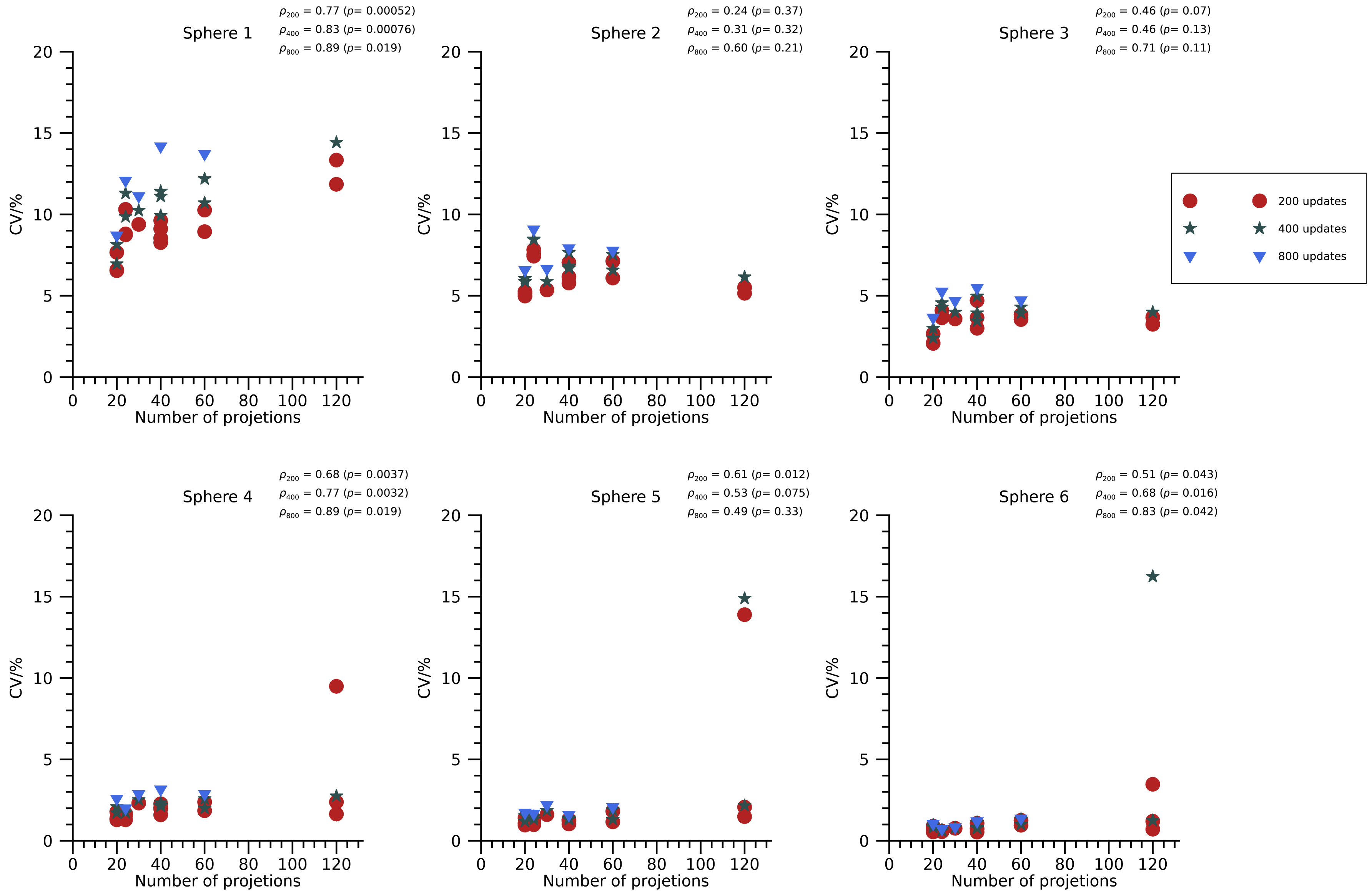


Figure S8: Coefficient of variation error as function of number of projections of the NEMA phantom measurements with a total acquisition time of 20 min. Results are shown for 200, 400, and 800 updates. Note that the ordinate range is individual for each plot, but, if necessary, has been capped at 20 %.

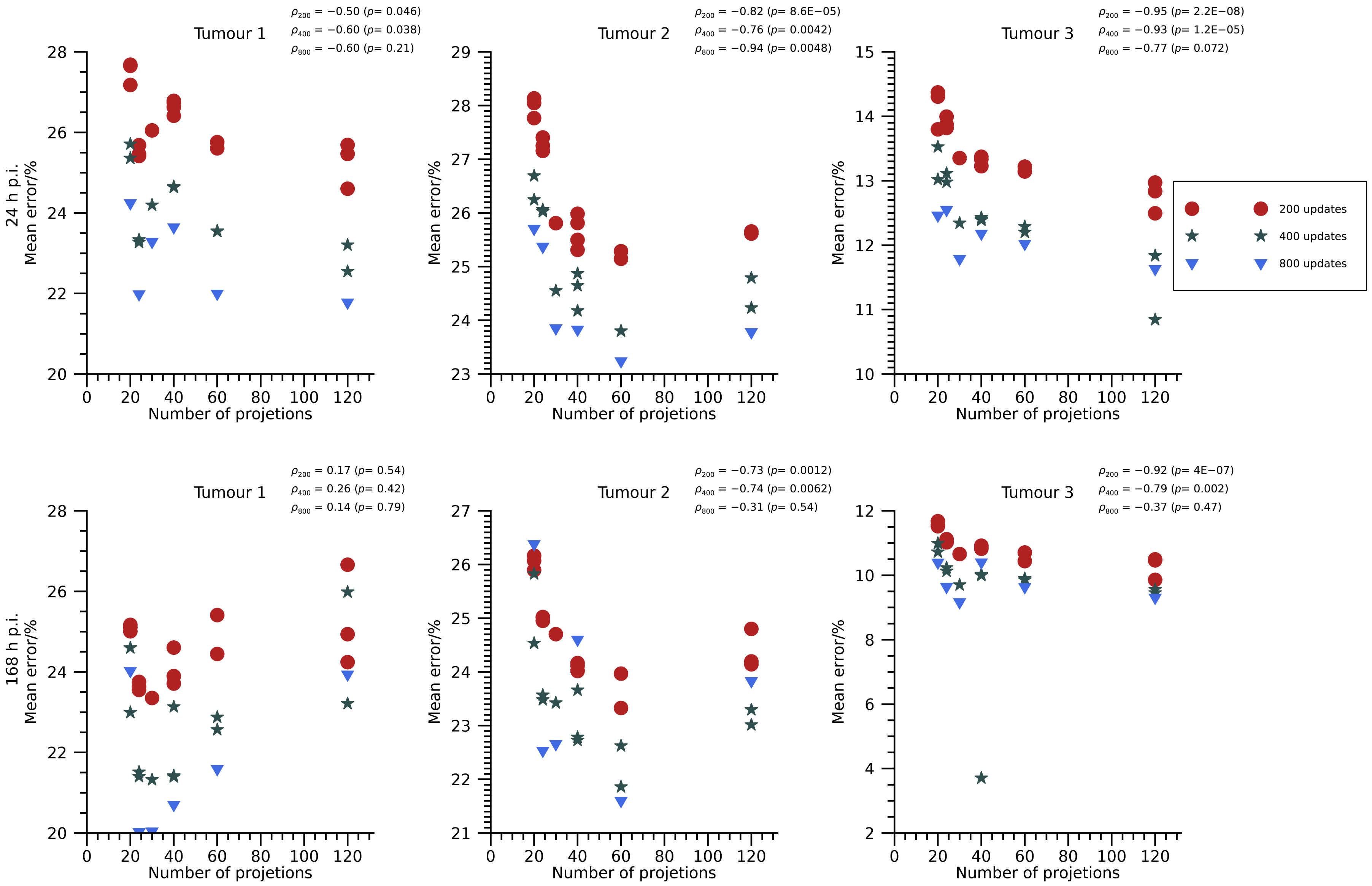


Figure S9: Absolute mean relative error as function of number of projections for the Monte Carlo simulations with a total acquisition time of 40 min. Results for images corresponding to 24 h p.i. are shown in the upper row and results corresponding to 168 h p.i. are shown in the lower row. Note that the ordinate range is individual for each plot.

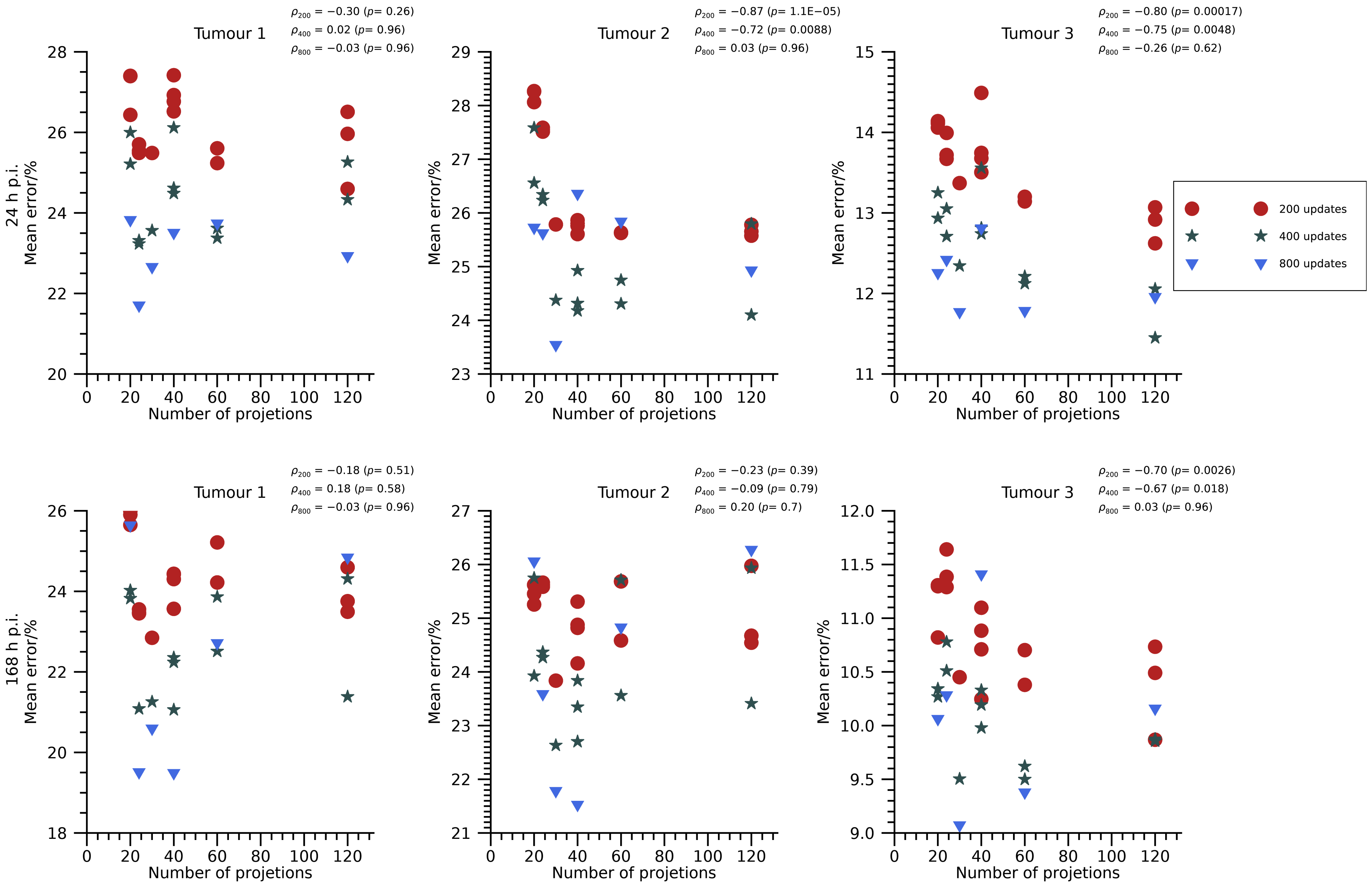


Figure S10: Absolute mean relative error as function of number of projections for the Monte Carlo simulations with a total acquisition time of 20 min. Results for images corresponding to 24 h p.i. are shown in the upper row and results corresponding to 168 h p.i. are shown in the lower row. Note that the ordinate range is individual for each plot.

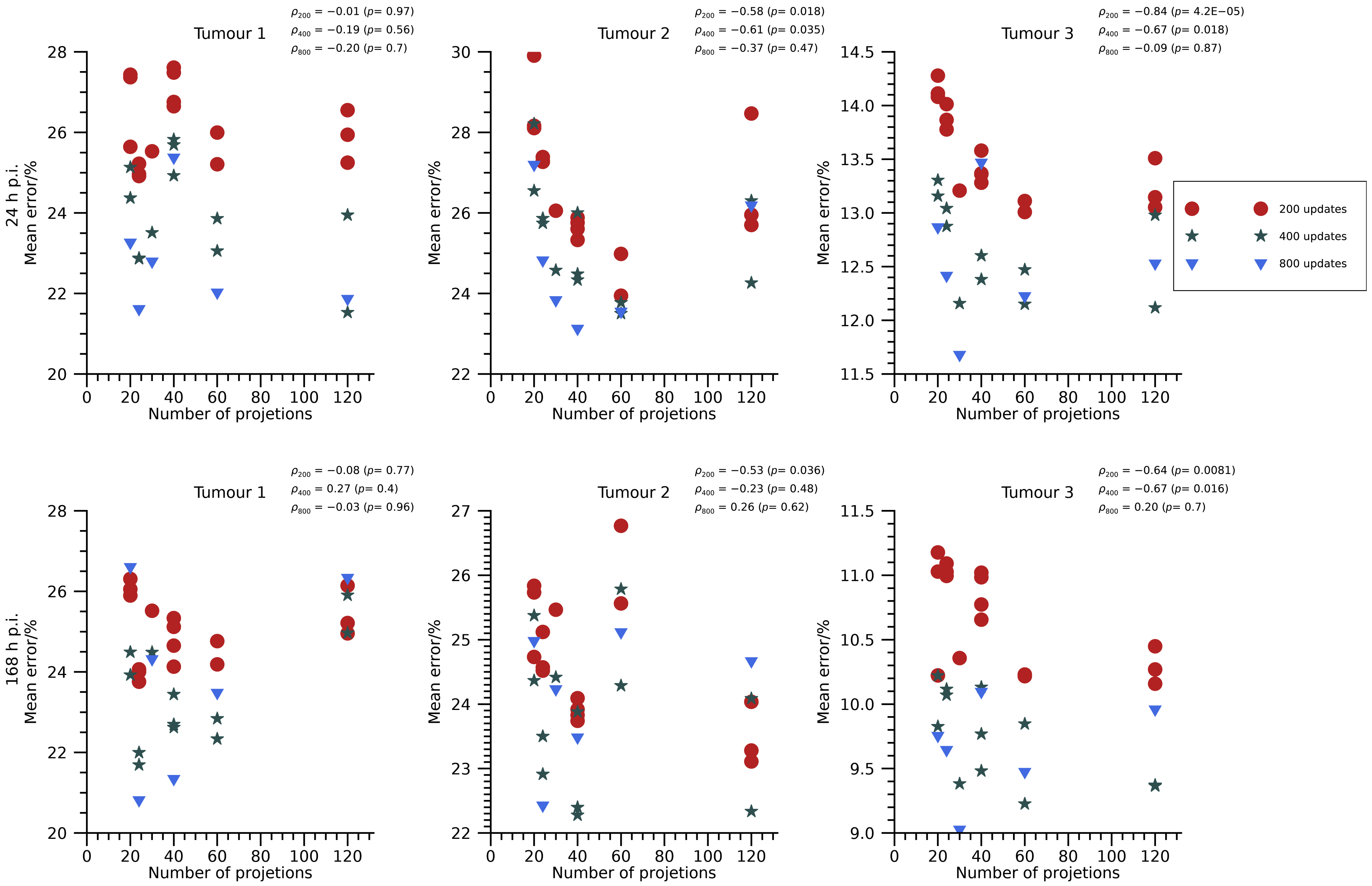


Figure S11: Absolute mean relative error as function of number of projections for the Monte Carlo simulations with a total acquisition time of 10 min. Results for images corresponding to 24 h p.i. are shown in the upper row and results corresponding to 168 h p.i. are shown in the lower row. Note that the ordinate range is individual for each plot.

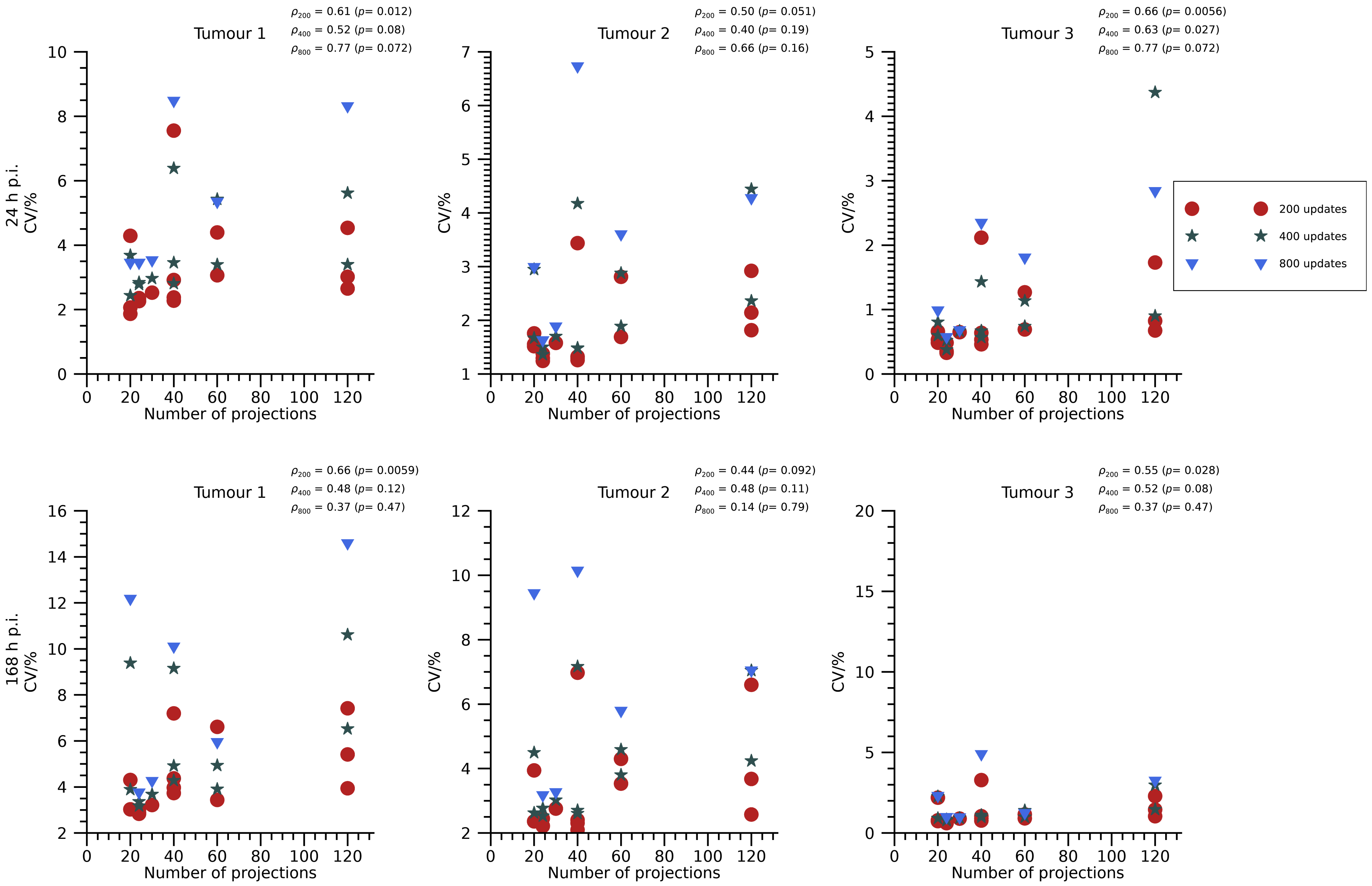


Figure S12: Coefficient of variation as function of number of projections for the Monte Carlo simulations with a total acquisition time of 40 min. Results for images corresponding to 24 h p.i. are shown in the upper row and results corresponding to 168 h p.i. are shown in the lower row. Note that the ordinate range is individual for each plot but, if necessary, has been capped to 20 %.

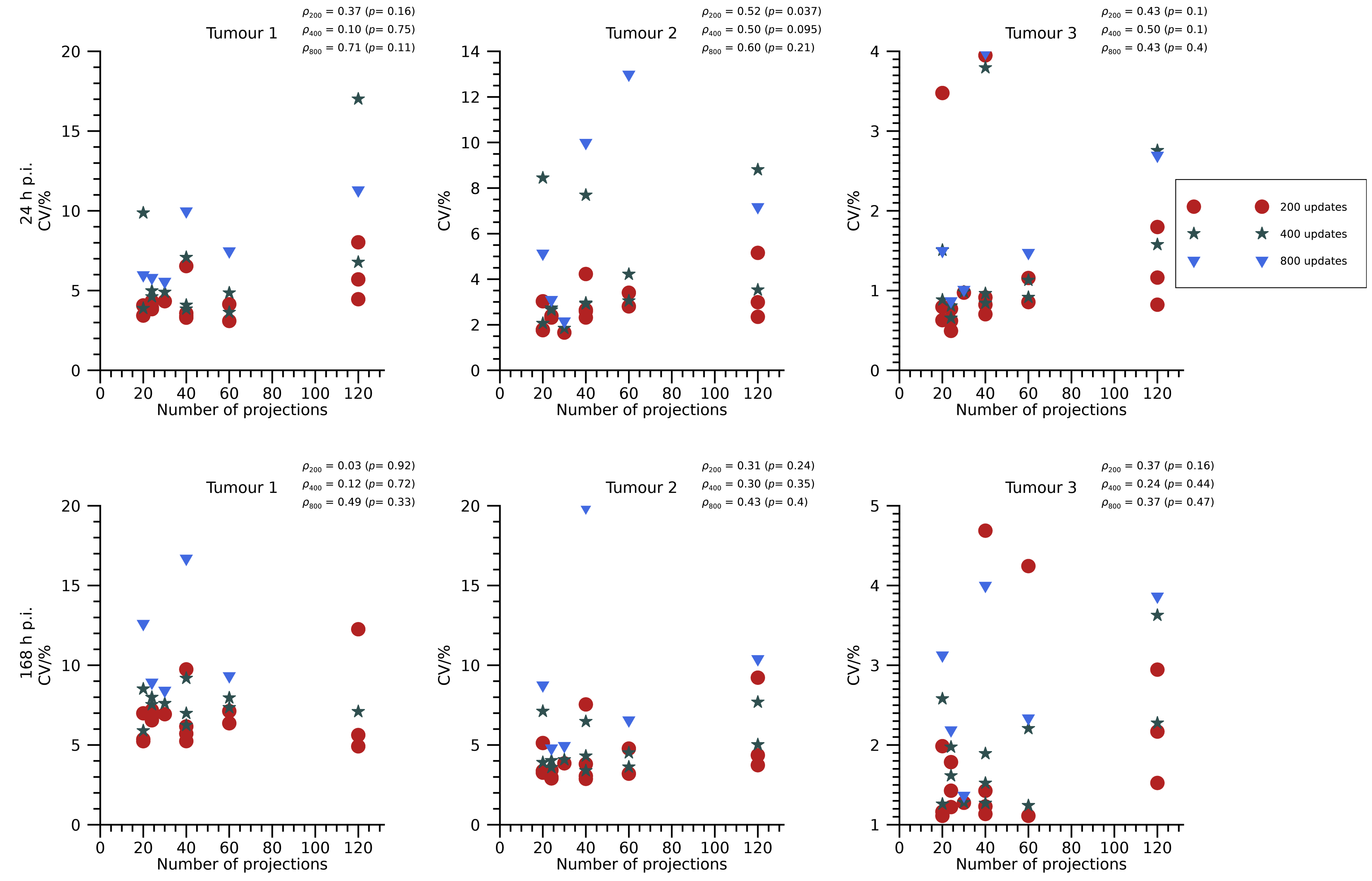


Figure S13: Coefficient of variation as function of number of projections for the Monte Carlo simulations with a total acquisition time of 20 min. Results for images corresponding to 24 h p.i. are shown in the upper row and results corresponding to 168 h p.i. are shown in the lower row. Note that the ordinate range is individual for each plot but, if necessary, has been capped to 20 %.

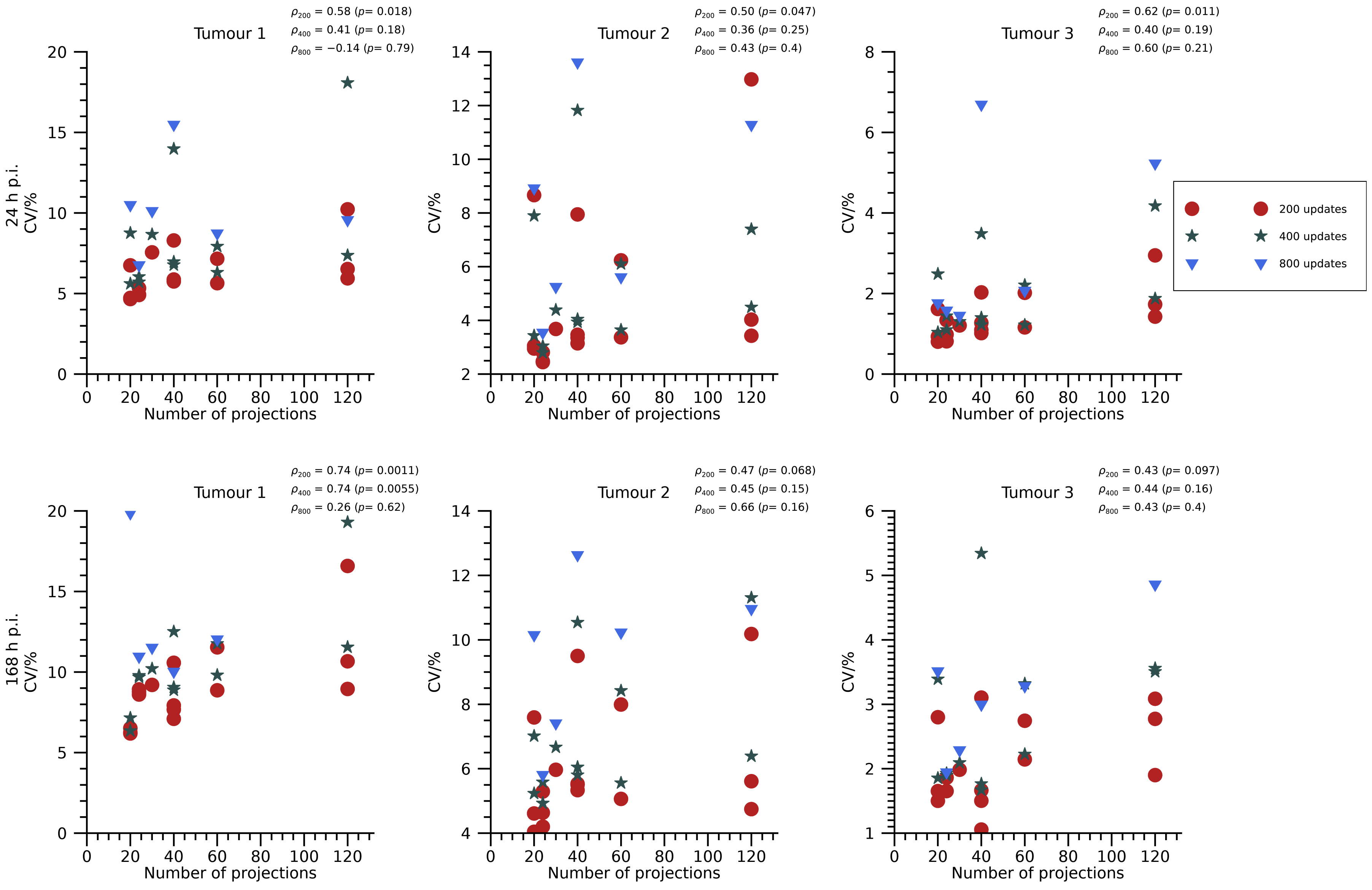


Figure S14: Coefficient of variation as function of number of projections for the Monte Carlo simulations with a total acquisition time of 10 min. Results for images corresponding to 24 h p.i. are shown in the upper row and results corresponding to 168 h p.i. are shown in the lower row. Note that the ordinate range is individual for each plot but, if necessary, has been capped to 20 %.

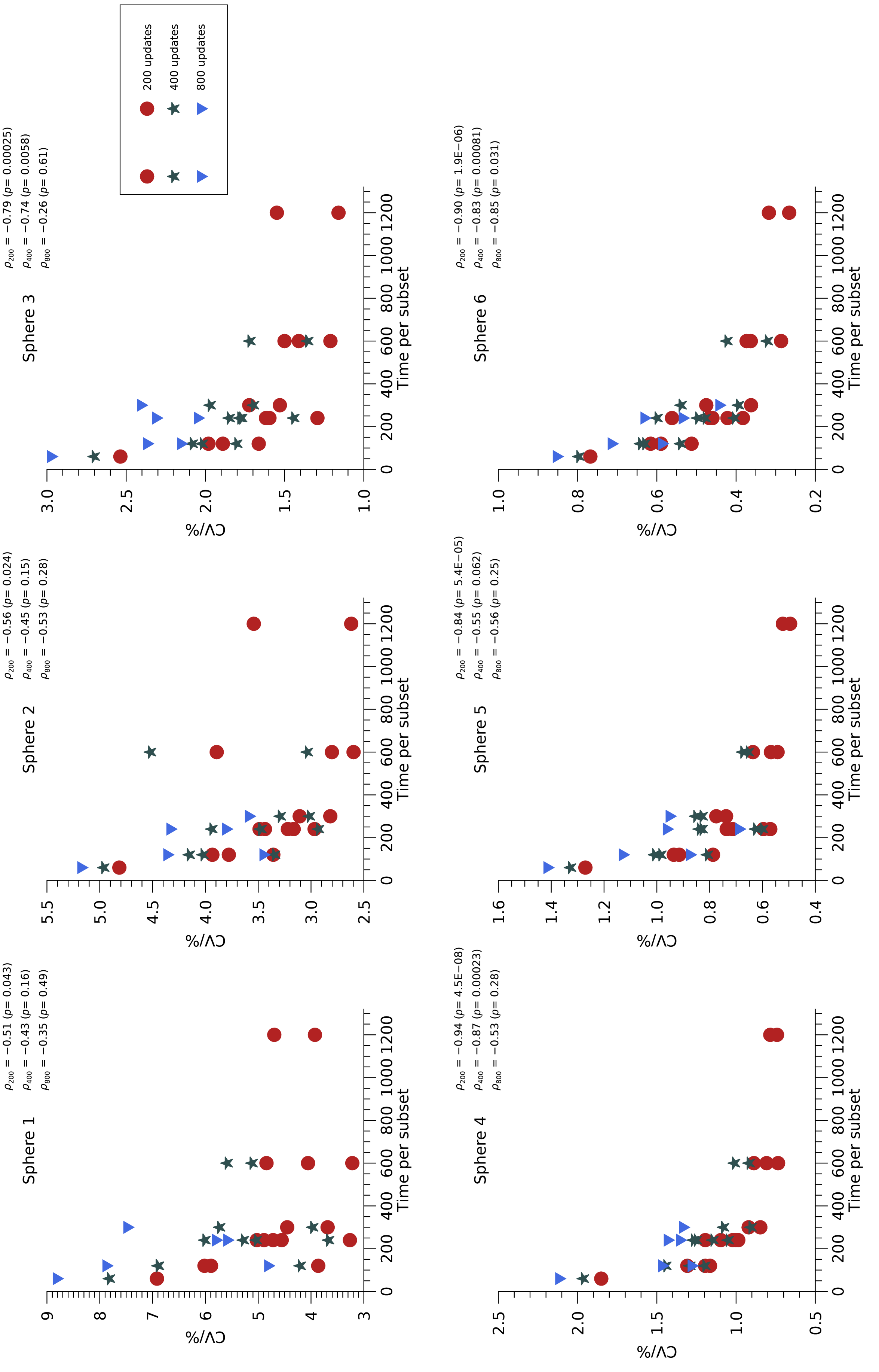


Figure S15: Coefficient of variation as function of time per subset for the NEMA phantom measurements with a total acquisition time of 40 min. Results are shown for 200, 400, and 800 updates. Note that the ordinate range is individual for each plot, but has, if necessary, been capped at 20 %.

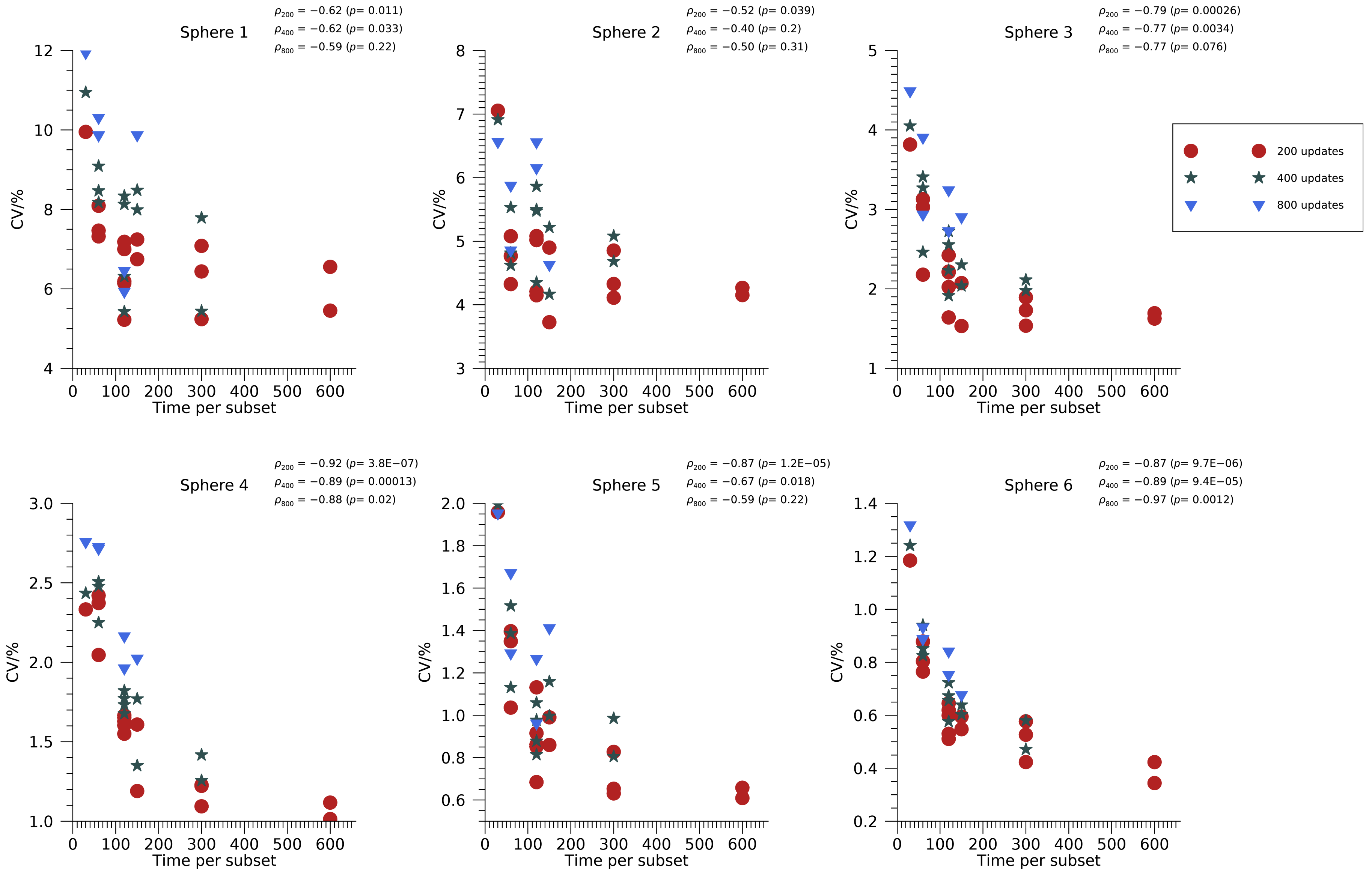


Figure S16: Coefficient of variation as function of time per subset for the NEMA phantom measurements with a total acquisition time of 20 min. Results are shown for 200, 400, and 800 updates. Note that the ordinate range is individual for each plot, but has, if necessary, been capped at 20 %.

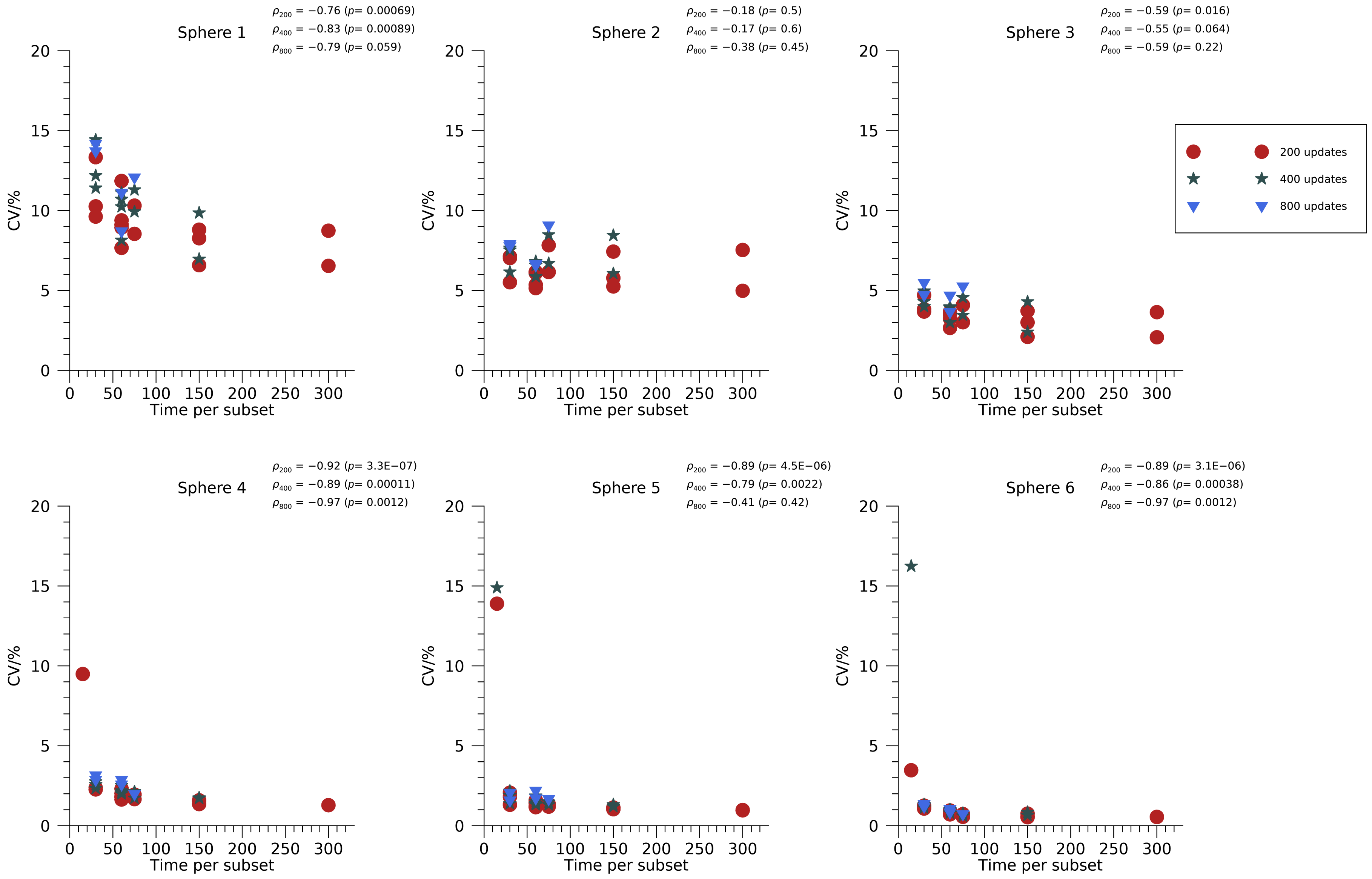


Figure S17: Coefficient of variation as function of time per subset for the NEMA phantom measurements with a total acquisition time of 10 min. Results are shown for 200, 400, and 800 updates. Note that the ordinate range is individual for each plot, but has, if necessary, been capped at 20 %.

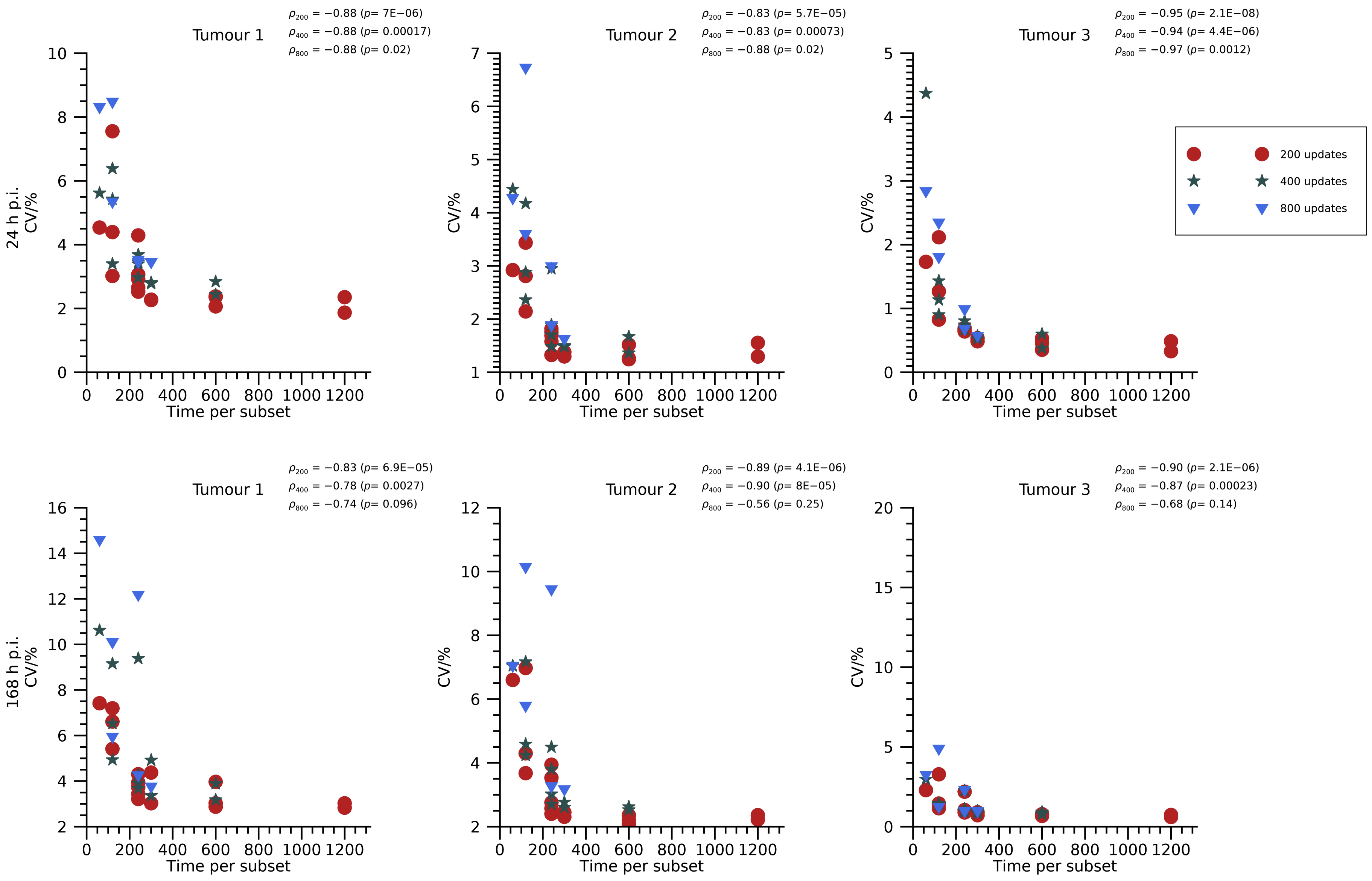


Figure S18: Coefficient of variation as function of time per subset for the Monte Carlo simulations with a total acquisition time of 40 min. Results for images corresponding to 24 h p.i. are shown in the upper row and results corresponding to 168 h p.i. are shown in the lower row. Note that the ordinate range is individual for each plot, but has, if necessary, been capped at 20 %.

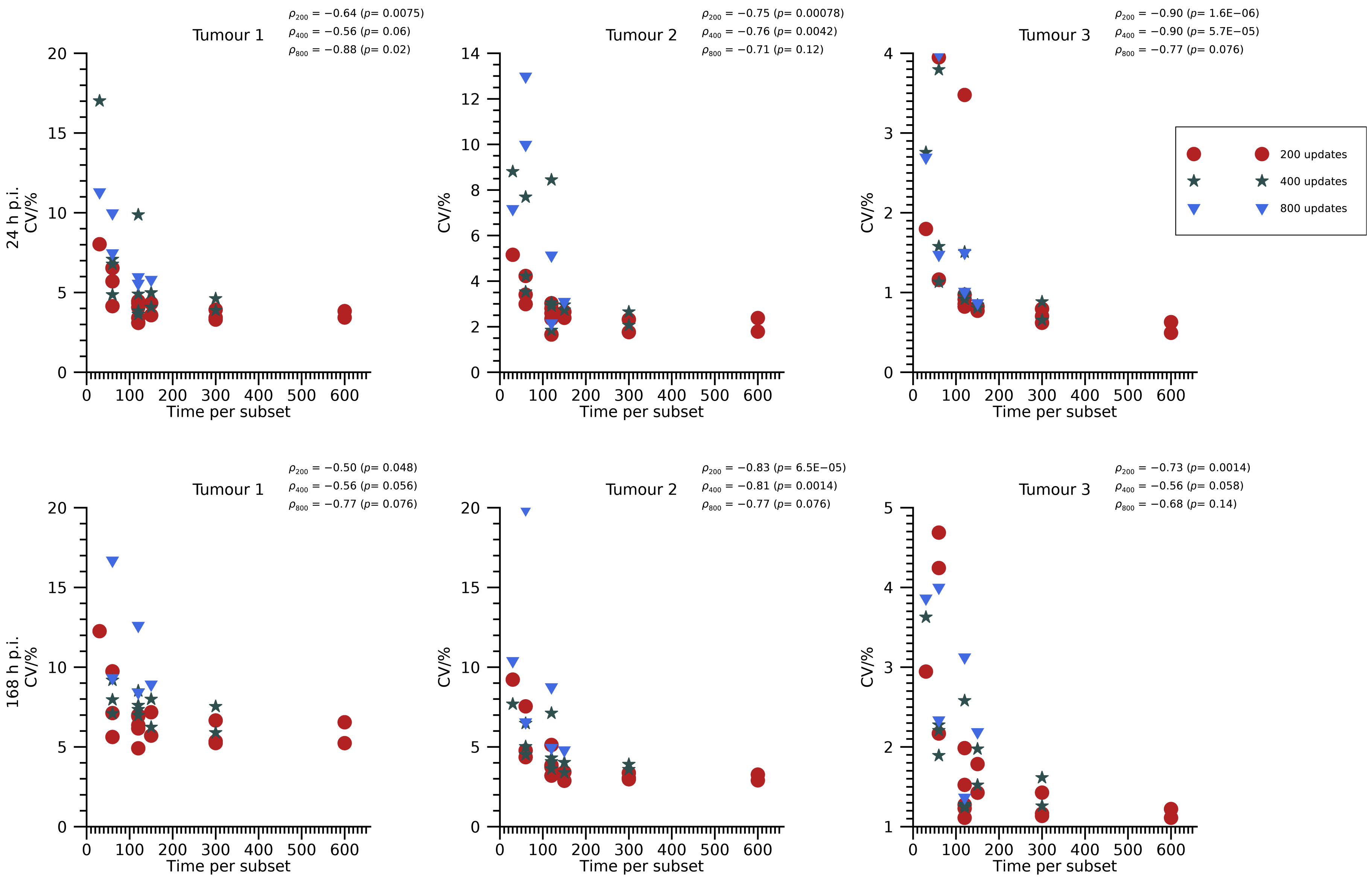


Figure S19: Coefficient of variation as function of time per subset for the Monte Carlo simulations with a total acquisition time of 20 min. Results for images corresponding to 24 h p.i. are shown in the upper row and results corresponding to 168 h p.i. are shown in the lower row. Note that the ordinate range is individual for each plot, but has, if necessary, been capped at 20 %.

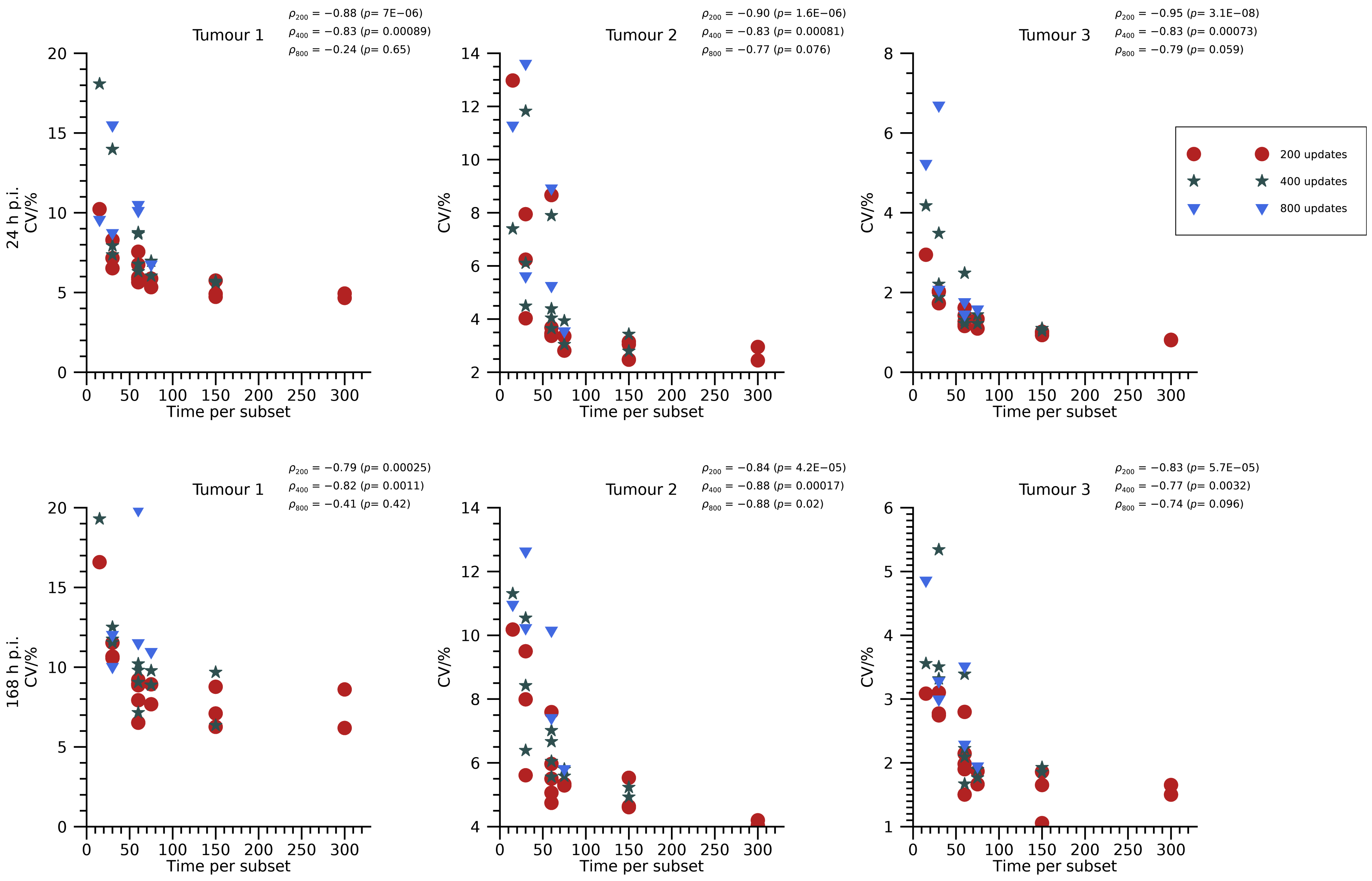


Figure S20: Coefficient of variation as function of time per subset for the Monte Carlo simulations with a total acquisition time of 10 min. Results for images corresponding to 24 h p.i. are shown in the upper row and results corresponding to 168 h p.i. are shown in the lower row. Note that the ordinate range is individual for each plot, but has, if necessary, been capped at 20 %.
